# YOLOv13-ADR: An Adaptive Deformable Convolution and Neighborhood-Aware Recombination Network for Wind Turbine Blade Defect Detection

**DOI:** 10.3390/s26165111

**Published:** 2026-08-12

**Authors:** Xinwei Wang, Muhammad Moman Shahzad, Shixuan Yang, Tianlong Wang, Zhihao Wang

**Affiliations:** 1 School of Civil Engineering and Communication, North China University of Water Resources and Electric Power, Zhengzhou 450045, China; wangxinwei@ncwu.edu.cn; 2 Department of Civil Engineering, National University of Sciences and Technology, Balochistan Campus, Quetta 86000, Pakistan; m.momin@nbc.nust.edu.pk; 3 Kang-Ju Construction Parts Certification Center, Centre of Science and Technology Industrial Development, Ministry of Housing and Urban-Rural Development of the People’s Republic of China, Beijing 100835, China; yangsxuan@stu.hqu.edu.cn; 4 CSCEC City Construction Development Co., Ltd., Beijing 100037, China; wtlong@mail.nwpu.edu.cn

**Keywords:** health monitoring, wind turbine blades, surface defect detection, YOLOv13, deformable convolution, failure analysis

## Abstract

Accurate detection of surface defects in wind turbine blades is critical for condition monitoring and preventive maintenance of wind energy systems. Defects such as cracks, burns, deformation, and peeling are characterized by small dimensions, irregular morphologies, and low contrast, limiting the effectiveness of conventional feature extraction methods. Although YOLOv13 enhances high-order feature correlation and information flow, its fixed-grid spatial sampling and content-agnostic upsampling operations remain limited in adapting to irregular defect geometries and preserving fine-grained boundary information. This study proposes YOLOv13-ADR, an enhanced detection framework integrating Adaptive Deformable Convolution (ADConv) and a Nearest Neighbor Content Perception Recombination (NNCPR) module. ADConv applies a geometry-driven kernel permutation strategy to strengthen multi-scale feature representation, while NNCPR improves neighborhood-aware perception for modeling geometric deformations. A Focus-IoU loss function incorporating an anchor-quality perception mechanism is introduced to accelerate training convergence and improve bounding box regression precision. Additional optimizations include modifications to the DS-C3k2 module and upsampling strategy. Experiments on a wind turbine blade defect dataset demonstrate that YOLOv13-ADR achieves a 7.26-percentage-point improvement in mean average precision over YOLOv8n, with enhanced small-defect recognition and reduced localization errors, demonstrating improved detection precision and localization performance relevant to early fault detection and structural health monitoring of wind turbine blades.

## 1. Introduction

Wind energy has become a major contributor to global renewable energy capacity, with progressively larger rotor diameters and blade lengths increasing the operational complexity and maintenance demands of wind turbines [1,2,3,4]. Wind turbine blades are subjected to fatigue loads, lightning strikes, and erosion, and are a significant contributor to overall turbine cost, making robust defect detection critical for operational safety and failure prevention. The remote locations and scale of wind farms make manual inspection costly and hazardous, motivating automated, vision-based defect detection as a research priority [5].

Offshore wind turbine blades are exposed to combined hydrodynamic, aerodynamic, and structural loading, and blade degradation directly threatens structural integrity. Operations and maintenance (O&M) costs account for 25–30% of total offshore lifecycle costs, with blade failures a leading contributor [6]. Automated defect detection supports condition-based maintenance, helping operators optimize inspection scheduling under constrained weather windows and vessel availability [7,8].

The proposed framework can also serve as a data layer for digital twin architectures, where high-fidelity detection outputs support real-time structural condition assessment [9,10]. This contributes to the broader shift from reactive to predictive offshore maintenance, reducing unplanned downtime.

Currently, due to the powerful advantages of deep learning algorithms in feature extraction and mining, they have achieved good application results in structural damage recognition, fault diagnosis [11,12,13], and defect detection. The defect detection method for wind turbine blades based on deep learning models can be divided into two architectures: two-stage object detection and single-stage object detection. The two-stage architecture delivers precise localization through region proposal and classification workflows: Faster R-CNN achieves 58% mean average precision at 4.9 frames per second [14], while cascade R-CNN further improves detection quality through a multi-stage architecture [15]. Encoder–decoder architecture provides unsupervised anomaly detection capabilities by learning latent representations of normal blade states, effectively identifying deviations in composite structures [16,17]. Generative adversarial networks (GANs) address the critical challenge of limited annotated data through synthetic defect generation, enhancing training dataset diversity and model robustness [18]. Hybrid Informer models leverage complex spatiotemporal features for dynamic modeling, demonstrating enhanced performance in intricate defect pattern recognition [19]. Single-stage detectors, particularly the You Only Look Once (YOLO) series, have emerged as dominant solutions by performing unified localization and classification within a singular detection paradigm. Compared to two-stage detectors, enhanced YOLO variants exhibit superior real-time performance [20,21,22,23,24]. Zhang et al. [25] proposed a lightweight YOLOv5s-based model for wind turbine surface defect detection, improving blade inspection accuracy. Li et al. [26,27,28] integrated unmanned aerial vehicle technology with deep learning algorithms to implement precise segmentation, detection, and tracking of wind turbine blades, enabling non-contact, non-destructive, cost-effective, and high-precision condition monitoring. Despite these advances, YOLO-based methods still exhibit insufficient feature extraction capabilities for fine-grained defect identification [29,30], particularly in distinguishing critical structural damage from surface anomalies under variable environmental conditions. Enhanced multi-scale feature extraction and fusion methodologies are consequently required to resolve the inherent trade-off between detection granularity and inference efficiency.

The adaptability of YOLO series algorithms to irregular defect shapes is improved by enhancing their ability to extract local features, thereby achieving the goal of improved defect detection accuracy based on the YOLO algorithms. Deformable convolution presents a promising approach for multi-scale feature mining [31,32,33], with its core advantage lying in liberating feature extraction from the rigid constraints of fixed geometric structures inherent in conventional kernels [34]. This mechanism enables networks to autonomously learn spatial offsets of sampling locations. Such dynamic adjustment capability liberates feature extraction from predefined rectangular grid patterns [35], establishing an adaptive paradigm where convolution kernel sampling points are dynamically repositioned according to local content, geometric deformations, scale variations, and complex poses within input data [36,37]. Despite deformable convolution’s capacity for kernel resizing, critical challenges persist in effectively uncovering latent multi-scale features and reorganizing neighborhood-aware multi-scale convolutional characteristics, which remain fundamental constraints for deep feature excavation.

To address the irregular geometries and non-uniform damage boundaries exhibited by wind turbine blade defects, an algorithm is proposed in this study wherein convolutional kernel sampling points are dynamically initialized according to geometric features and adaptively repositioned, thus enabling enhanced extraction of multi-scale object features. Furthermore, dual-branch architecture is designed to dynamically generate content-aware upsampling kernels while adaptively aggregating neighborhood features. This effectively resolves non-integer coordinate interpolation issues in adaptive convolution and strengthens feature representation capabilities. An adaptive penalty factor based on target dimensions and an anchor quality perception mechanism are developed: the former dynamically adjusts regression weights for small targets to mitigate gradient imbalance, while the latter suppresses gradient interference from low-quality anchors. These innovations demonstrate superior efficacy to traditional loss functions in scenarios involving small-object detection within dense environments while simultaneously expediting the training process. High-precision recognition of wind turbine blade defects is achieved through three key advancements: deep multi-scale feature excavation, adaptive neighborhood feature aggregation, and innovatively constructed loss functions.

The principal contributions of this work include the following:

(i) Existing convolutional architectures are constrained to fixed, regular kernel geometries, limiting their ability to represent the irregular and non-uniform boundaries characteristic of blade surface defects. We address this by proposing an adaptive deformable convolution (ADConv) algorithm that removes the fixed-kernel-dimension constraint, enabling arbitrary kernel configurations with task-specific initial sampling distributions and near-square convergence-optimized geometries.

(ii) Adaptive convolution methods that predict sampling offsets inherently produce non-integer sampling coordinates, a longstanding interpolation problem that existing upsampling approaches address only through content-agnostic interpolation (e.g., bilinear), degrading boundary fidelity for small targets. We resolve this with a nearest neighbor content perception recombination (NNCPR) module that generates content-aware upsampling kernels, enabling accurate multi-scale feature reorganization without the boundary degradation associated with conventional interpolation.

(iii) Standard IoU-based loss functions apply uniform regression penalties regardless of target scale or anchor quality, causing gradient imbalance that disproportionately harms small-object detection, a critical limitation for defects such as fine cracks. We address this with a Focus-IoU (F-IoU) loss function that introduces an anchor-quality perception mechanism to suppress gradient contributions from low-quality anchors, combined with a scale-adaptive penalty factor, jointly accelerating convergence and improving regression accuracy for small and multi-scale defects.

These three contributions address three distinct limitations in existing detection frameworks. First, fixed-geometry convolutional kernels cannot represent the irregular, non-uniform boundaries characteristic of blade surface defects; ADConv addresses this through geometry-driven permutation initialization combined with learnable sampling offsets, enabling arbitrary kernel dimensions and adaptive receptive field shaping. Second, adaptive sampling mechanisms such as ADConv inherently generate non-integer sampling coordinates, and conventional upsampling operations such as bilinear interpolation are content-agnostic and degrade boundary fidelity for small targets; NNCPR addresses this through content-aware upsampling kernel prediction and nearest-neighbor feature recombination. Third, standard IoU-based loss functions apply uniform regression penalties regardless of target scale or anchor quality, causing gradient imbalance that disproportionately affects small-object detection; F-IoU addresses this through a scale-adaptive penalty factor combined with an anchor-quality perception mechanism. Section 3.1, Section 3.2 and Section 3.3 detail each of these components in turn.

This paper is organized as follows. Section 2 introduces the basic theory of YOLOv13. Section 3 presents the proposed ADConv, NNCPR, F-IoU, and YOLOv13-ADR framework. Section 4 describes the construction and processing of WTDD. Section 5 presents the experimental results and discussion, including comparative, sampling-configuration, generalization, and ablation experiments. Section 6 concludes the study, and Section 7 discusses the limitations and future work.

## 2. Basic Theory

### 2.1. YOLOv13

YOLOv13 is employed as the principal network framework in this research. In comparison to alternative models within the YOLO series [38,39,40,41], the distinctiveness of YOLOv13 is marked by the incorporation of a Hypergraph-based Adaptive Correlation Enhancement (HyperACE) and a Full-Pipeline Aggregation-and-Distribution (FullPAD), which facilitate the adaptive investigation of high-order pixel correlations while concurrently optimizing fine-grained information flow and collaborative representation learning. Furthermore, an efficient feature extraction module has been developed utilizing depthwise separable convolution, wherein traditional wide-kernel convolution modules are substituted with depthwise separable counterparts, thereby enabling the achievement of accelerated inference speeds.

#### 2.1.1. HyperACE

**Figure 1:**
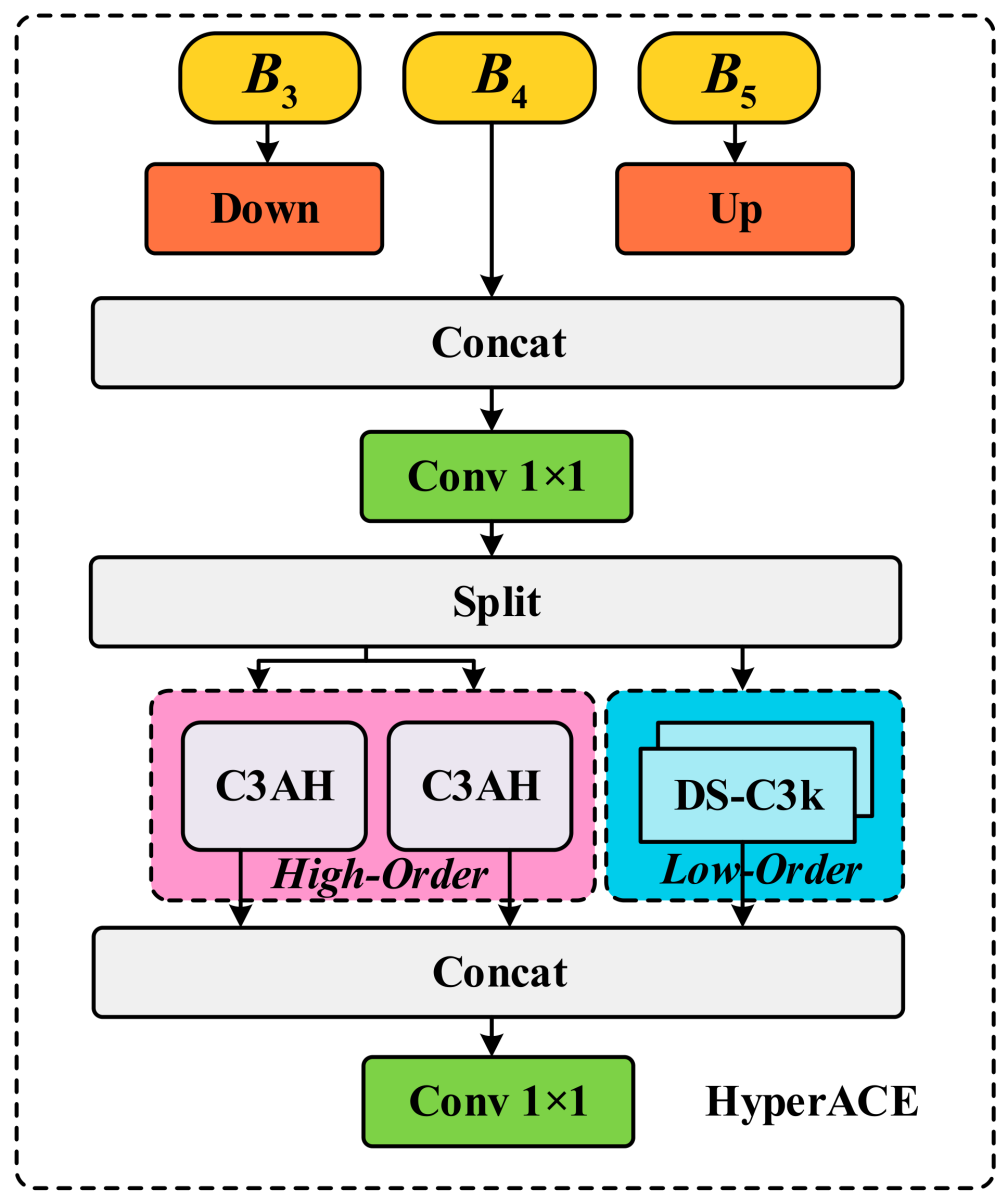
Framework of HyperACE.

To address the limitations associated with manual parameter configuration in traditional hypergraph methods [42], a learnable hyperedge generation module is designed within HyperACE. Through this module, hyperedge construction is adaptively learned and executed based on input visual features, enabling the dynamic exploration of latent inter-vertex relationships across feature spaces. By overcoming previous constraints in pairwise correlation modeling, efficient global cross-position and cross-scale feature fusion and enhancement are achieved through integrated hierarchical representation learning. The framework of HyperACE is shown in Figure 1.

Specifically, $X=x_{i}\in C,i=1,2,\cdot \cdot \cdot ,N$ represents vertex features, where *C* is the number of feature channels. Define context vectors $f_{avg}$ and $f_{\max}$ through global average pooling and max pooling, respectively, and concatenate them to obtain global vertex context $f_{ctx}$. This process can be expressed as:

(1) $$f_{ctx}=\left(\begin{array}{l} f_{avg}\\ f_{\max} \end{array}\right)\in 2C$$

Next, the global offset is generated from the vertex context through the mapping layer and added to the learnable global prototype to obtain the hyperedge prototype $P_{m}$. To further enhance the diversity of features, a multi-head mechanism is introduced to divide the vertex query vector $z_{i}$ into h subspaces ${\left\{{\hat{z}}_{i}^{\tau }\in d_{h}\right\}}_{\tau =1}^{h}$ along the feature $z_{i}=W_{pre}x_{i}$, where $W_{pre}$ is the weight matrix, $d_{h}=C/h$. Divide each hyperedge prototype into subspaces ${\left\{{\hat{P}}_{m}^{\tau }\in d_{h}\right\}}_{\tau =1}^{h}$. Subsequently, in the subspace τ, the similarity between the i-th vertex query vector and the hyperedge can be calculated using the following equation.

(2) $$s_{i,m}^{\tau }=\frac{\langle {\hat{z}}_{i}^{\tau },{\hat{p}}_{m}^{\tau }\rangle }{\sqrt{d_{h}}}$$

The overall similarity ${\tilde{s}}_{i}$ is subsequently defined as the average similarity computed across all subspaces. The similarity between the vertex query vector and the prototype is adopted as the measure of vertex contributions to hyperedges, with normalization being performed across the vertex dimension. The continuous participation matrix $A_{i,m}$ can then be derived using the following formulation:

(3) $$A_{i,m}=\frac{\exp({\tilde{s}}_{i},m)}{\sum_{j=1}^{N}\exp({\tilde{s}}_{j,m})}$$

After generating adaptive hyperedges, perform hypergraph convolution to achieve feature aggregation and enhancement.

(4) $$f_{m}=\sigma \left(W_{e}\sum_{i=1}^{N}A_{i,m}\cdot x_{i}\right)$$

(5) $${\tilde{x}}_{i}=\sigma \left(W_{v}\sum_{m=1}^{M}A_{i,m}\cdot f_{m}\right)$$

Among them, *i* is the vertex index and m is the hyperedge index. *I* and $W_{v}$ represent the projection weights of hyperedges and vertices, respectively. σ is the activation function.

#### 2.1.2. FullPAD

FullPAD is designed to collect multi-scale feature maps from backbone networks and input them into HyperACE. Through the utilization of distinct FullPAD channels, enhanced features are dynamically redistributed to targeted positions within the channel architecture [43]. This paradigm is not only instrumental in achieving fine-grained information flow and collaborative representation but also contributes significantly to improved gradient propagation efficiency and enhanced detection performance. Specifically, after the correlation-enhanced feature Y is generated by HyperACE at stages B3, B4, and B5, spatial resolution adjustment is performed by FullPAD across each channel, while channel dimensionality is modified through a 1 × 1 convolutional layer. This operational sequence is formally expressed as:

(6) $$H_{i}=Conv_{1\times 1}(\mathrm{Resize}(Y\leftarrow size(B_{i}))),i\in \left\{3,4,5\right\}$$

Then, for the feature map $F_{i}$ of any stage i, gate fusion is used to achieve information flow fusion.

(7) $${\hat{F}}_{i}=F_{i}+\gamma H_{i}$$

Among these components, γ is introduced as a learnable scalar parameter dedicated to the adaptive mediation of contribution weighting between correlation-enhanced features and original features.

## 3. Proposed Novel Network Model

To address the irregular geometries of wind turbine blade defects, the susceptibility of boundary details to information loss, and the imbalance of regression gradients for small objects, this study proposes ADConv, NNCPR, and F-IoU. ADConv jointly adjusts the convolutional receptive field through initial sampling topology design and dynamic offset learning. Centered on non-integer deformed coordinates, NNCPR performs neighborhood localization, content-aware recombination, and cross-sampling-point aggregation. F-IoU redistributes regression gradients according to object scale and anchor quality. The three modules operate sequentially at the stages of feature sampling, feature reconstruction, and bounding-box optimization, thereby forming a detection pipeline with explicit inter-module dependencies rather than a simple combination of mutually independent components.

### 3.1. Adaptive Deformable Convolution

As introduced in Section 1, conventional deep neural networks are constrained by fixed-configuration convolutional grids, limiting model adaptability to diverse targets. We propose adaptive deformable convolution, which supports arbitrary kernel configurations (e.g., 5 × 5, 6 × 6, 7 × 7) through a geometry-driven permutation initialization algorithm and a learnable offset mechanism. This allows kernels to dynamically reshape their receptive fields according to input feature geometry, improving multi-scale feature extraction for geometrically diverse objects.

Unlike conventional deformable convolutions, which predict sampling offsets from a fixed regular grid, ADConv first generates a configurable initial spatial topology according to the number of sampling points and then further adjusts each sampling position using independently learned offsets. Consequently, the number of sampling points, their initial arrangement, and their final spatial distribution can all be adaptively varied, eliminating the constraint imposed by fixed square convolutional grids. This mechanism establishes morphology-specific receptive fields for defects such as elongated cracks, localized burn, and irregular deformation, thereby extending deformable convolution from positional deformation over a fixed topology to joint deformation of the initial topology and sampling positions.

To enable irregular convolution operations, a novel geometry-driven sampling topology generation algorithm is implemented in the proposed ADConv method for constructing spatial configuration patterns of sub-kernels. This three-step algorithmic procedure commences with the establishment of a regular grid benchmark, followed by the introduction of a controlled irregular deformation mechanism, and culminates in optimized sampling point configuration through grid fusion. Specifically, the algorithm has the following characteristics:

(1) Initialization coordinate generation is supported by convolution kernels of arbitrary dimensions.

(2) Algorithmic universality is maintained while square-approximated sampling distributions are prioritized to optimize network convergence.

(3) The top-left kernel position is designated as the origin (0,0), contrasting with conventional center-based coordinate systems where the convolution centroid is conventionally defined as the origin. This approach is demonstrated to be more effective for handling coordinate uniformity across variably sized kernels, thereby establishing a robust foundation for subsequent adaptive deformation.

Initially, the permutation base value b and row count r are derived from the convolutional kernel dimension N, upon which a canonical permutation grid $Array_{i\times j}^{reg}$ is systematically constructed. This computational procedure is formally expressed by Equation (8).

(8) $$Array_{i\times j}^{reg}=\left\{i,j\left|i\in [0,r-1]\right.,j\in [0,b-1]\right\}$$

Among them, $b=Round(\sqrt{N})$, $r=\left\lfloor \frac{N}{b}\right\rfloor$, $\left\lfloor \cdot \right\rfloor$ represent rounding downwards.

Subsequently, the residual sub-kernel count m is computed, followed by the construction of an irregular configuration grid $Array_{r\times j}^{irreg}$ for these residual elements. This algorithmic procedure is formally defined by:

(9) $$Array_{i\times j}^{irreg}=\left\{r,j\left|j\in [0,m-1]\right.\right\}\;(m=N\mathrm{mod}\;b)$$

The initial arrangement position $P_{N}$ of the complete convolution kernel obtained by concatenating regular and irregular grids can be represented as:

(10) $$P_{N}=Array_{i\times j}^{reg}\cup Array_{r\times j}^{irreg}$$

According to the different sizes of convolution kernels, the permutation initialization algorithm can generate initial sampling permutation shapes for convolution operations of any size.

**Figure 2:**
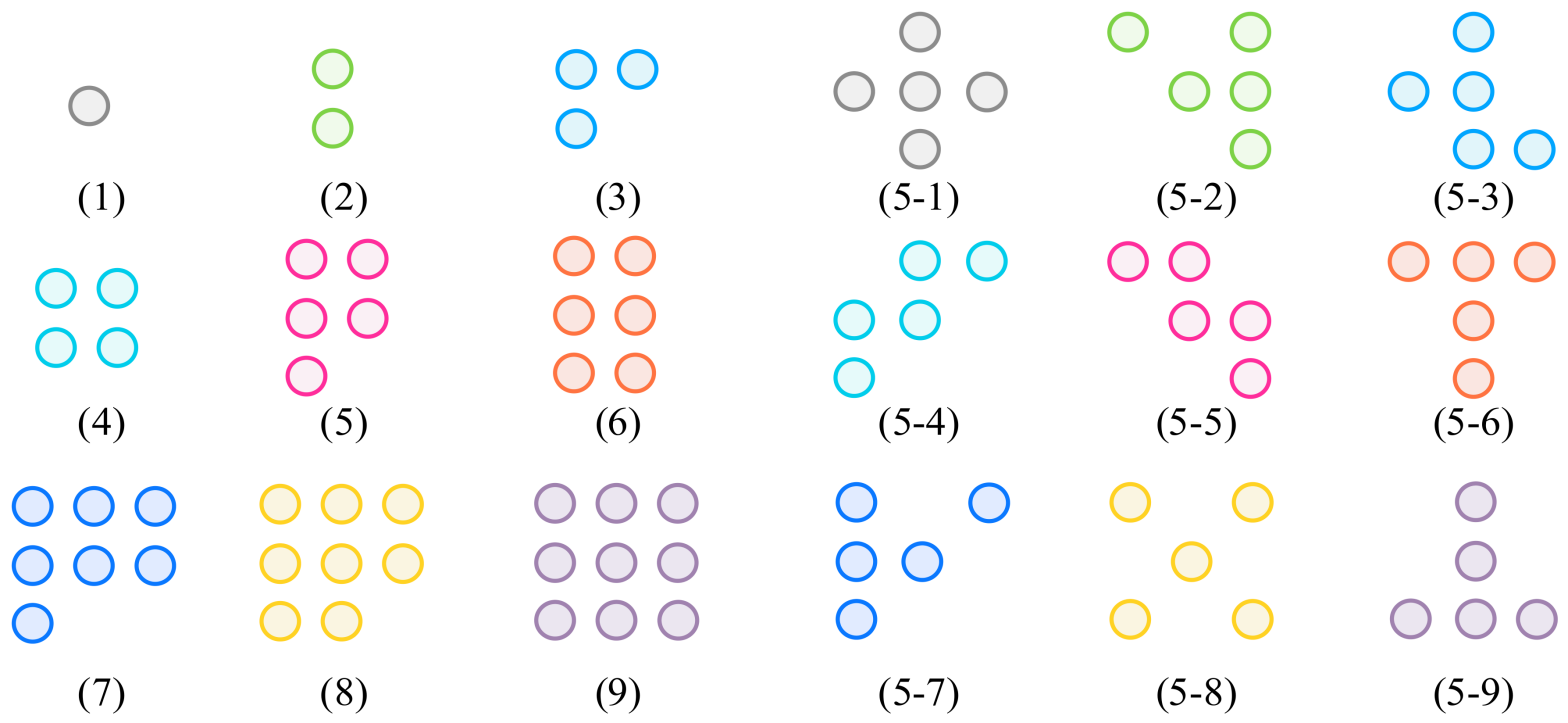
Initial sampling shape and shape-adaptive adjustment of convolution kernels of different sizes.

The permutation initialization algorithm proposed in this work adaptively generates initial sampling point distributions for convolution operations of arbitrary dimensions based solely on kernel size parameter N. As illustrated in Figure 2, support is established not only for conventional kernel sizes (e.g., N = 3, 5, 7), but crucially, effective implementation is achieved for non-standard kernel dimensions. Taking N = 5 as representative case, an initial sampling grid is first constructed by ADConv, after which sampling point positions are dynamically adjusted through a learnable offset mechanism. This enables convolutional kernels to adapt their spatial configuration according to target geometric characteristics. Empirical exploration of sampling shapes demonstrates that this deformable paradigm significantly enhances kernel adaptability to heterogeneous targets, particularly for objects exhibiting complex geometries. Following offset optimization, multiple refined sampling modes are formed, thereby enabling precise capture of discriminative target features.

After constructing the initial sampling shape of the convolutional kernel, the input image features will be fed into the ADConv proposed in this paper for offset adjustment. The entire process adopts cascaded architecture, first dynamically adjusting the sampling point positions through learnable offsets, then implementing content aware feature recombination based on the nearest neighbor content aware recombination module and finally completing multi-scale feature extraction. This progressive feature processing mechanism enables the final obtained enhanced features to have geometric adaptability, and their receptive field can be dynamically adjusted according to the actual shape of the target object, thereby significantly improving the diversity and discriminability of feature expression. In terms of specific implementation, the offset prediction module is constructed using a lightweight Conv2d (1 × 1) convolutional layer, which can directly act on the input feature map to generate an offset Δp that is strictly aligned with the spatial dimension of the feature map. Its mathematical expression can be described as:

(11) $$\Delta p=Conv_{2d}(X,\theta_{input})=Offset(X,\theta_{offset})$$

Among them, $\theta_{input}$ is the dimension (B,C,H,W) of the input image. $\theta_{offset}$ is the output dimension (B,2N,H,W). B is the batch size, C is the number of channels, and H and W represent the height and width of the feature map, respectively.

The offset feature map generated by the 1 × 1 convolution has 2*N* channels, since each of the *N* sampling points requires two independent offset parameters (Δx, Δy) for its x- and y-axis displacement. This allows each sampling point to adjust its position independently based on the local content of the input feature map, enabling the kernel to adapt to the target’s geometric deformation. For example, an offset of (Δx, Δy) at a given position shifts the corresponding sub-kernel by that amount. Through backpropagation, the network learns to predict offsets that align the kernel’s spatial distribution with the target’s geometry, improving robustness to deformation. The optimized convolution kernel space arrangement is denoted as $P_{offset}$, and its corresponding convolution operation can be calculated according to Equation (12).

(12) $$P_{offset}=P_{N}+\Delta p$$

(13) $$Conv(P_{offset})=\sum_{P_{N}\in Array}\omega_{p_{n}}\times x(P_{N}+\Delta p)$$

Among them, Array, $\omega_{p_{n}}$ and $x(P_{N}+\Delta p)$ respectively represent the generated sampling grid, convolution parameters, and pixel values at corresponding positions.

ADConv comprises four sequential operations: initial coordinate generation, offset prediction, feature resampling, and convolutional aggregation. Given an input feature map $X\in {\mathbb{R}}^{B\times C\times H\times W}$, the initial coordinates $P_{N}$ are first generated according to the number of sampling points *N*. Subsequently, a 1 × 1 Conv2d layer with a stride of 1 maps the input features to an offset tensor with 2*N* channels, where each pair of channels represents the horizontal and vertical displacements of one sampling point. The resulting sampling positions are denoted by $P=P_{0}+P_{N}+\Delta P$. After neighborhood-based feature resampling, the sampled features are reshaped to B × *C* × *N* × *H* × *W*. A Conv3d operation with both the kernel size and stride set to (*N*,1,1) is then applied along the sampling-point dimension for feature aggregation, followed sequentially by batch normalization and SiLU activation. This produces an output feature map of size *B* × *C_out_* × *H* × *W*. The proposed structure preserves the spatial resolution of the input features while enabling each spatial location to learn an independent adaptive sampling geometry. The pseudocode (Algorithm 1) of the Adaptive Deformable Convolution algorithm is shown below.
**Algorithm 1:** Adaptive Deformable Convolution**Input:** Input feature map F∈B,C,H,W, Kernel size *N***Output:** Output feature map $F\in {\mathbb{R}}^{B\times C_{\mathrm{out}}\times H\times W}$1: Calculate *b* and *r* according to kernel size: $b\leftarrow round(\sqrt{N})$, $r\leftarrow \left\lfloor N/b\right\rfloor$2: Layout $A_{reg}$ by Equation (8) $\leftarrow \{(i,j)\left|i\in [0,r-1]\right|,\left|j\in [0,b-1]\right|\}$3: Layout $A_{irreg}$ by Equation (9) $\leftarrow \{(r,j)\left|j\in [0,m-1]\right|,\left|j\in [0,b-1]\right|\}$4: Initial grid $A_{N}\leftarrow A_{reg}\cup A_{irreg}$5: Calculate the predicted offset Δp by Equation (11)6: Calculate deformable grid $A_{\mathrm{def}}$ by Equation (12)7: **For** each $A\in A_{\mathrm{def}}$ **do:**8: Feature extraction by Equation (13)←Conv(A)9: $Y\leftarrow \;Y+\omega \cdot x\;(A_{N}+\Delta p)$10: **End for**11. **Return**
F

### 3.2. Nearest Neighbor Content Perception Recombination Module

As introduced in Section 1, adaptive sampling mechanisms such as ADConv generate non-integer sampling coordinates. The Nearest Neighbor Content Perception Recombination Module (NNCPR) is proposed to address this issue. This module adopts a dual-branch architecture design, where the upsampling kernel prediction unit is responsible for dynamically generating content-aware upsampling kernels. The nearest neighbor content recombination unit then adaptively aggregates features within the neighborhood of the sampling points based on the predicted kernel weights. This mechanism resolves the interpolation problem at non-integer coordinates and improves feature representation by fusing multi-scale context information. The NNCPR module significantly improves the model’s feature extraction ability for geometrically deformed targets while maintaining the continuity of the feature space, providing more discriminative feature representations for subsequent network layers.

Unlike conventional upsampling methods, such as nearest-neighbor interpolation, bilinear interpolation, and transposed convolution, NNCPR does not rely on fixed mapping relationships or spatially shared convolution kernels. Instead, for each output location, NNCPR maps the location to the corresponding continuous coordinate in the low-resolution feature map and performs content-aware recombination within its local neighborhood. The corresponding nearest integer position and its *k* × *k* neighborhood are first determined. Location-specific recombination kernels are then dynamically generated according to the local feature content and used to perform weighted aggregation of the neighborhood features. On this basis, NNCPR further jointly enhances the *N* groups of deformed features along the sampling-point dimension, thereby unifying non-integer coordinate mapping, neighborhood-content restoration, and multi-sampling-point feature fusion within a single computational pipeline.

First, given a feature map X of size (C,H,W) and an upsampling ratio δ, NNCPR will output a new feature map $X^{\prime }$ of size (C,δ×H,δ×W) to expand perceptual information. Therefore, for any pixel position ${ο}^{\prime }(x^{\prime },y^{\prime })$ in the output feature map $X^{\prime }$, its corresponding pixel position in the original feature map X is ο(x,y), where $x=\left\lfloor x^{\prime }/\delta \right\rfloor$, $y=\left\lfloor y^{\prime }/\delta \right\rfloor$. If the non-integer sampling coordinate ο(x,y) is taken as the center, then its nearest neighbor and k × k coordinate regions located in integer coordinates are taken as its neighbors, denoted as $O(X_{ο(x,y)},k)$.

After determining the neighboring region, the upsampling kernel prediction unit ψ(⋅) will predict a perceptual recombination kernel $\zeta_{{ο}^{\prime }(x^{\prime },y^{\prime })}$ for each pixel position. This process can be expressed as:

(14) $$\zeta_{{ο}^{\prime }(x^{\prime },y^{\prime })}=\psi (O(X_{ο(x,y)},k))$$

Each pixel position in the original feature map corresponds to $\delta^{2}$ pixel positions in the reconstructed feature map. Each pixel position in the feature map requires a *k* × *k* perceptual recombination kernel, so the size of the upsampling kernel output by this unit is $\left(\delta^{2}\times k^{2},H,W\right)$.

Specifically, the upsampling kernel prediction unit includes three submodules: channel compressor, content encoder, and kernel normalizer. Firstly, the channel compressor uses 1 × 1 convolution to reduce the feature map channel count from C to $C_{0}$, improving computational efficiency. Next, based on the input features, the content encoder uses a convolutional layer with a kernel size of k to generate a perceptual recombination kernel. The larger the value of k, the larger the perception area of the perceptual recombination kernel, and the content of the adjacent area obtained is rich. However, the computational complexity will increase with the square of the size of the perceptual recombination kernel. Finally, each *k* × *k* sized perceptual recombination kernel will be spatially normalized using the Softmax function. The normalization operation forces the sum of kernel values to be 1, as a soft selection across adjacent regions.

For each perceptual recombination nucleus, the nearest neighbor content recombination unit will reorganize the features of the adjacent area through the function φ(⋅). This process can be expressed as:

(15) $$X^{\prime }=\varphi (O(X_{ο(x,y)},k),\zeta_{{ο}^{\prime }(x^{\prime },y^{\prime })})$$

Among them, φ(⋅) represents the calculation of weighted sum.

Therefore, for the pixel position center ${ο}^{\prime }(x^{\prime },y^{\prime })$ and the corresponding adjacent area $O(X_{ο(x,y)},k)$, the final reorganized feature map can be expressed as:

(16) $$X^{\prime }=\sum_{a=-\frac{k}{2}}^{\frac{k}{2}}\sum_{b=-\frac{k}{2}}^{\frac{k}{2}}{ο}^{\prime }(x^{\prime },y^{\prime })\cdot X_{ο(x+a,y+b)}$$

For perceptual recombination kernels, the contribution of each pixel within the $O(X_{ο(x,y)},k)$ region to the up-sampled pixel center ${ο}^{\prime }(x^{\prime },y^{\prime })$ is different, depending on the content of the features. The restructured feature map combines relevant pixel information from neighboring regions, resulting in a more comprehensive aggregation of upper and lower level information, and is more refined compared to the original feature map.

**Figure 3:**
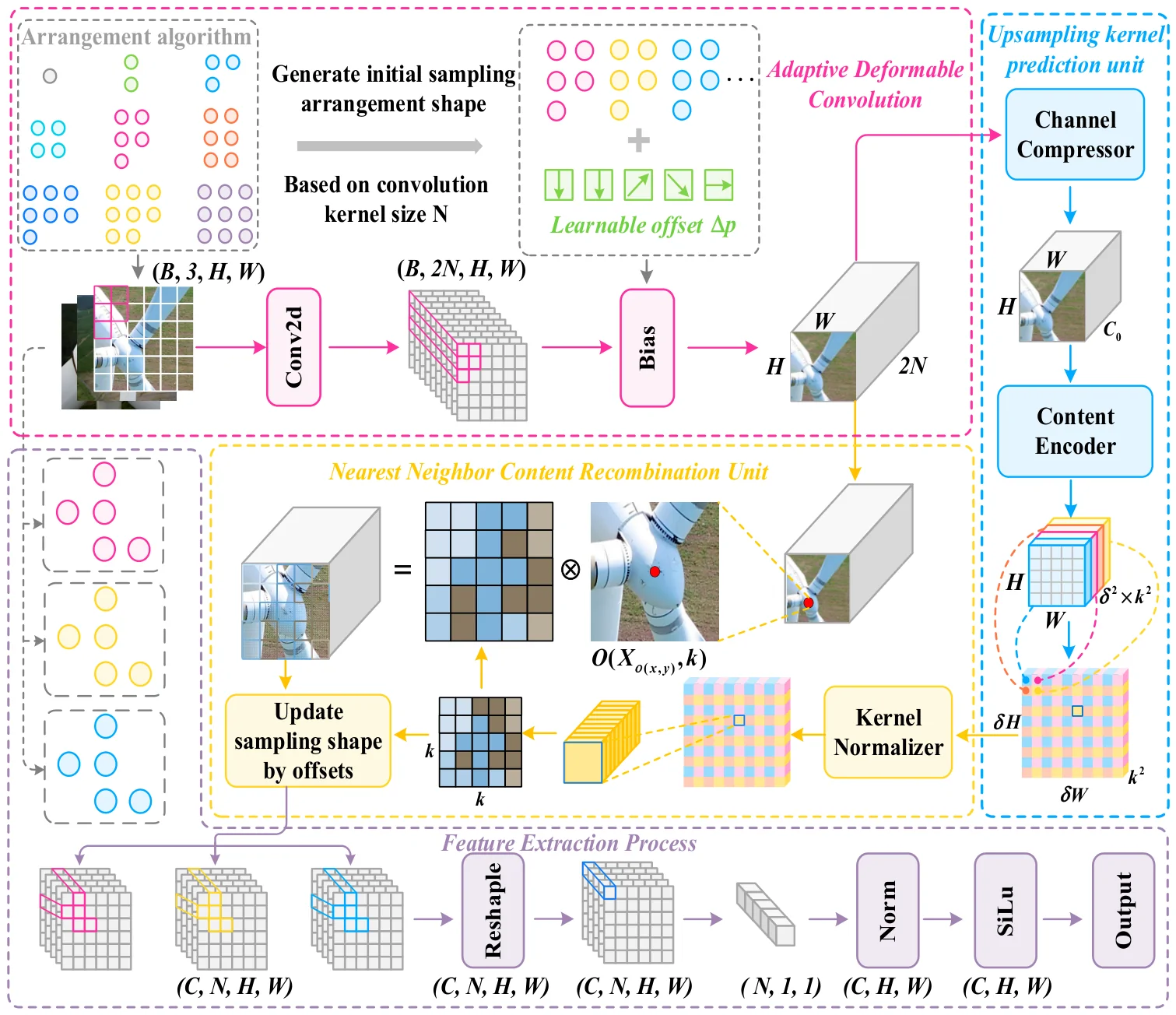
Framework of ADConv and NNCPR modules.

After completing feature recombination, a convolution kernel adaptively adjusted through geometric deformation is used to extract deep features from the recombined features. Subsequently, a Conv3d operation with a parameter of (N,1,1) is used to perform multi-scale feature enhancement on the feature map after sampling position update and feature recombination. This cascaded feature processing flow not only effectively preserves the spatial structure information of the original features, but also further enhances the network’s feature representation ability for complex deformed targets through the spatiotemporal modeling capability of 3D convolution. Finally, after standardization and SiLU activation function, the final output Output(C,H,W) can be obtained. The architecture diagram of the ADConv and NNCPR modules proposed in this article is shown in Figure 3.

NNCPR consists of an upsampling-kernel prediction branch and a neighborhood-feature recombination branch. In this study, the upsampling factor is set to σ=2, and the lightweight configuration specified by $C_{m}=64$, $k_{encoder}=3$, and $k_{up}=5$ is adopted. In the kernel prediction branch, a 1 × 1 convolution first compresses the number of input channels from *C* to 64, after which a 3 × 3 content-encoding convolution generates a 100-channel output. This output is reshaped into four groups of 5 × 5 recombination kernels for each input location, and Softmax normalization is performed over the 25 neighborhood weights. In the feature recombination branch, a 5 × 5 neighborhood is extracted around the nearest integer location corresponding to each non-integer sampling coordinate. The neighborhood features are then aggregated through a weighted summation using the predicted kernel weights. Finally, the four outputs associated with each input location are rearranged along the spatial dimensions to obtain an upsampled feature map of size *B* × *C* × 2*H* × 2*W*. Since this process does not introduce cross-channel feature transformation, it preserves defect boundaries and local structural information with relatively low computational overhead. The pseudocode (Algorithm 2) for Nearest Neighbor Content Perception Recombination is as follows:
**Algorithm 2:** Nearest Neighbor Content Perception Recombination**Input:** Input feature map F∈B,C,H,W, Upsampling factor σ, Neighborhood size *k***Output:** Upsampling feature map F∈B,C,σH,σW1: **// upsampling Kernel Prediction Unit**2: Channel compressor$\leftarrow F\in B,C_{0},H,W$3: Define the neighbor region $O(X_{ο(x,y)},k)$ and origin ο(x,y)4: Output upsampling kernel$\leftarrow \zeta_{{ο}^{\prime }(x^{\prime },y^{\prime })}\in B,\delta^{2}\times k^{2},H,W$ by Equation (14)5: **// Nearest Neighbor Content Reorganization Unit**6: **For** each coordinate (*x*, *y*) **do:**7: Define the neighbor region of the coordinate (*x*, *y*)$\leftarrow O(X_{ο(x,y)},k)$8: Feature extraction by Equations (15) and (16)9: Output upsampling feature map F10: **End for**11. **Return**
F

### 3.3. Focus-IoU Loss Function

As introduced in Section 1, standard IoU-based loss functions apply uniform regression penalties regardless of target scale or anchor quality. Addressing this limitation, the present study introduces the Focus-IoU loss function, which significantly enhances bounding box regression performance through a dual optimization mechanism. First, it incorporates a target-size-based adaptive penalty factor that dynamically adjusts the penalty strength based on gradient for regression deviations according to target scale characteristics, effectively mitigating gradient imbalance in small object detection. Second, it integrates an anchor quality perception mechanism that evaluates the spatial relationships between anchor and ground-truth boxes. This mechanism adaptively suppresses the gradient contribution of low-quality anchors, reducing their negative impact on training. Compared to conventional loss functions like GIoU, CIoU, and WIoU, F-IoU demonstrates superior performance in detecting small objects and multi-scale dense targets while significantly accelerating training convergence.

Unlike GIoU and CIoU, which primarily improve geometric relationships by introducing constraints on the enclosing region, center distance, or aspect ratio, Focus-IoU specifically addresses the imbalance in gradient allocation across objects of different scales and anchors of varying quality. It constructs a scale-sensitive penalty based on the relative deviations between the four boundaries of the predicted and ground-truth boxes, such that the same absolute localization error is assigned a higher optimization weight for small objects. Meanwhile, an anchor-quality-aware mechanism suppresses the interfering gradients generated by low-quality samples that deviate substantially from the ground-truth boxes. Furthermore, FA-IoU employs a non-monotonic attention function to strengthen the learning of medium-quality anchors. Therefore, F-IoU integrates object-scale focusing, low-quality sample suppression, and medium-quality sample enhancement within a unified framework, rather than merely adding a single geometric constraint to an existing IoU-based loss.

In addition, variations in class frequency, object scale, and anchor quality can lead to an uneven distribution of effective regression gradients. To address this issue, F-IoU increases the optimization weight assigned to localization errors of small-scale objects through a scale-adaptive penalty and suppresses the interfering gradients generated by low-quality predictions through anchor-quality perception. Consequently, for small-scale defect categories with limited samples, F-IoU strengthens effective regression supervision and alleviates insufficient optimization caused by class imbalance. It should be noted that F-IoU primarily mitigates regression-gradient imbalance, whereas class-frequency imbalance is addressed using the class-aware repeat-sampling strategy described in Section 4.

**Figure 4:**
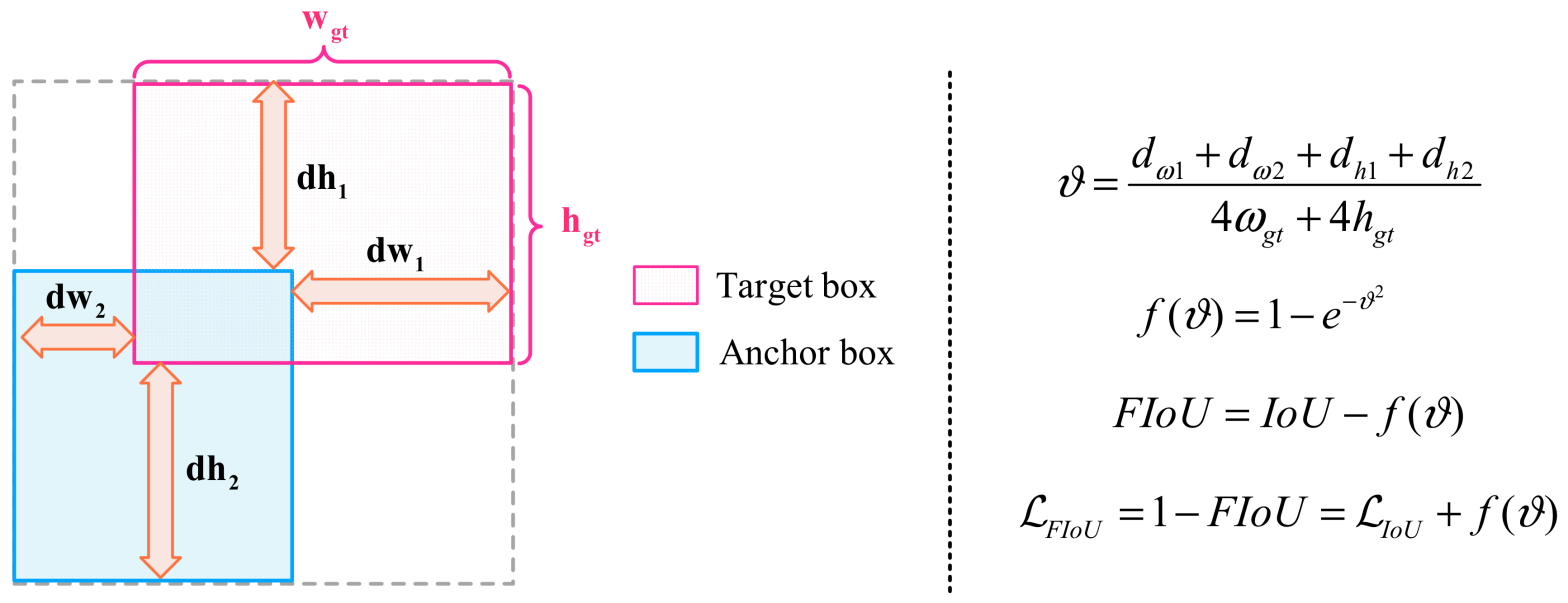
Definition of the penalty factor for F-IoU.

Specifically, the penalty factor is defined by multiple absolute distances in Figure 4 and is denoted as ϑ.

(17) $$\vartheta =\frac{d_{\omega 1}+d_{\omega 2}+d_{h1}+d_{h2}}{4\omega_{gt}+4h_{gt}}$$

Among them, $d_{\omega 1}$, $d_{\omega 2}$, $d_{h1}$, and $d_{h2}$ are the absolute distances from the predicted box and the target box to the four borders, respectively. $\omega_{gt}$ and $h_{gt}$ are the width and height of the target box, respectively.

The F-IoU loss function combines IoU loss with a penalty term ϑ based on penalty factors. The penalty term is adjusted through the function f(ϑ), which dynamically adjusts the gradient size based on the quality of the anchor box. The definition of the F-IoU loss function is as follows:

(18) FIoU=IoU−f(ϑ)

(19) $$L_{FIoU}=1-FIoU=L_{IoU}+f(\vartheta )$$

Among them, IoU represents the intersection and union ratio between the predicted box and the target box. f(ϑ) is a penalty item, specifically in the form of $f(\vartheta )=1-e^{-\vartheta^{2}}$. This function ensures that when ϑ is large (when the anchor box quality is poor), the gradient is small, thereby suppressing the harmful gradient caused by low-quality anchor boxes. When ϑ is small (i.e., anchor box quality is high), the gradient gradually decreases, allowing the anchor box to be stably optimized to be perfectly aligned with the target box.

In addition, F-IoU further enhances its focusing ability on medium quality anchor boxes by introducing a non-monotonic attention function. Firstly, the quality index of the anchor box is defined as A(λμ), the non-monotonic attention function is μ, and the F-IoU loss function introducing the non-monotonic attention function is FA-IoU. The definition of the FA-IoU loss function is as follows.

(20) $$A(\lambda \mu )=3(\lambda \mu )\cdot e^{-{\left(\lambda \mu \right)}^{2}}$$

(21) $$L_{FAIoU}=A(\lambda \mu )\cdot L_{FIoU}$$

Among them, λ is the hyperparameter that controls the attention function, $\mu =e^{-\vartheta }$. A(λμ) enables FA-IoU to focus more on medium quality anchor boxes, thereby improving the performance of the object detector.

### 3.4. Wind Turbine Blade Defect Detection Framework

Within the original YOLOv13 framework, the DS-C3k2 module primarily relies on static depthwise separable convolutions, which have limited adaptability to the irregular geometries of wind turbine blade defects. Consequently, fine-grained boundary information may be weakened, particularly for elongated cracks and locally deformed defects. In addition, the conventional interpolation-based upsampling operation does not explicitly account for feature semantics, potentially causing boundary blurring and insufficient contextual aggregation during multi-scale feature fusion.

**Figure 5:**
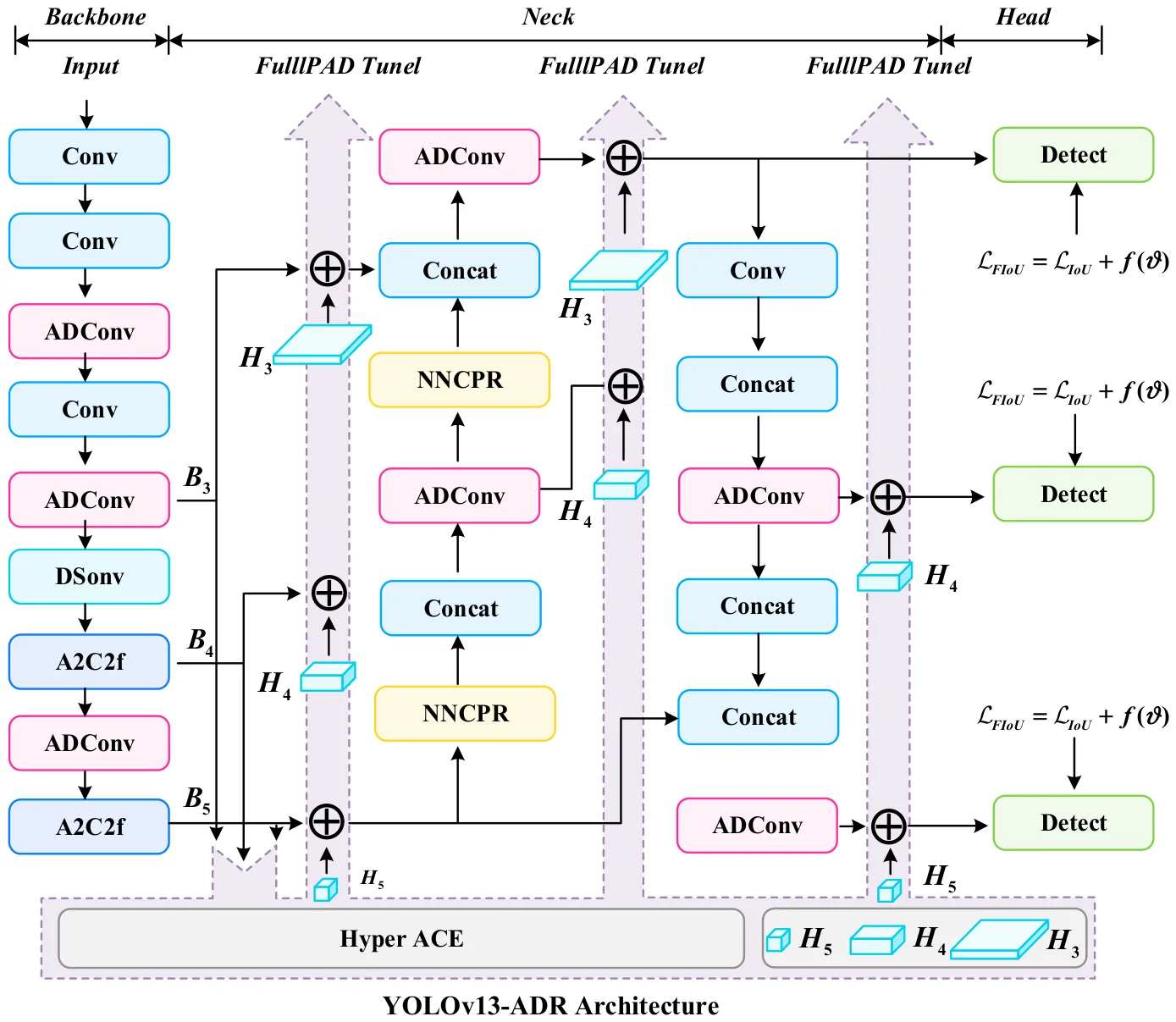
The framework of YOLOv13-ADR proposed in this study.

To address these limitations, YOLOv13-ADR integrates ADConv, NNCPR, and F-IoU into the feature extraction, feature fusion, and bounding-box regression stages, respectively. As illustrated in Figure 5, the original input stem, A2C2f modules, HyperACE modules, and three-scale detection heads of YOLOv13 are retained, while only the spatial feature extraction and upsampling operations are modified. At the B3, B4, and B5 stages of the backbone, the convolutions responsible for spatial feature extraction within the original DS-C3k2 modules are replaced with ADConv. The input and output channel dimensions, downsampling strides, and residual connection schemes at each stage remain unchanged.

In the top-down pathway of the neck, NNCPR with an upsampling factor of 2 is employed to perform the H5 → H4 and H4 → H3 scale transformations. The upsampled features are subsequently concatenated with backbone features of the same spatial resolution, after which ADConv is applied for geometry-adaptive refinement. The bottom-up pathway and the output resolutions of the H3, H4, and H5 detection heads remain unchanged. During training, F-IoU is incorporated into the bounding-box regression objective to redistribute regression gradients according to object scale and anchor quality, without introducing additional inference-time parameters.

The three components therefore form a progressively coupled optimization pipeline. ADConv first adapts the sampling topology and receptive field to the geometric characteristics of the target. NNCPR then reconstructs neighborhood context and fine-grained boundary information around the deformed sampling coordinates through content-aware feature recombination. Finally, F-IoU optimizes bounding-box localization by strengthening effective regression supervision for small-scale and difficult defects while suppressing interference from low-quality predictions. All newly introduced modules are jointly optimized with the original YOLOv13 architecture in an end-to-end manner. The overall architecture of YOLOv13-ADR is presented in Figure 5.

## 4. Wind Turbine Defect Data Acquisition and Processing

In this study, multiple UAV inspection sorties were conducted over a large-scale wind farm, yielding a total of 3251 original high-resolution images of wind turbine blades. The collected data contain seven representative defect categories: burn, crack, deformation, dirt, oil contamination, peeling, and rusty.

According to unified defect-category definitions, each visible defect instance was annotated with a rectangular bounding box using the Labeling tool and assigned the corresponding category label. The dataset contains a total of 18,479 defect instances, including 915 burn instances, 2208 cracks, 2006 deformations, 5829 dirt instances, 837 oil-contamination instances, 4856 peeling instances, and 1828 corrosion instances, as summarized in Table 1. Dirt and peeling account for relatively large proportions of the dataset, whereas oil contamination and burn are comparatively underrepresented, indicating a certain degree of class imbalance.

**Figure 6:**
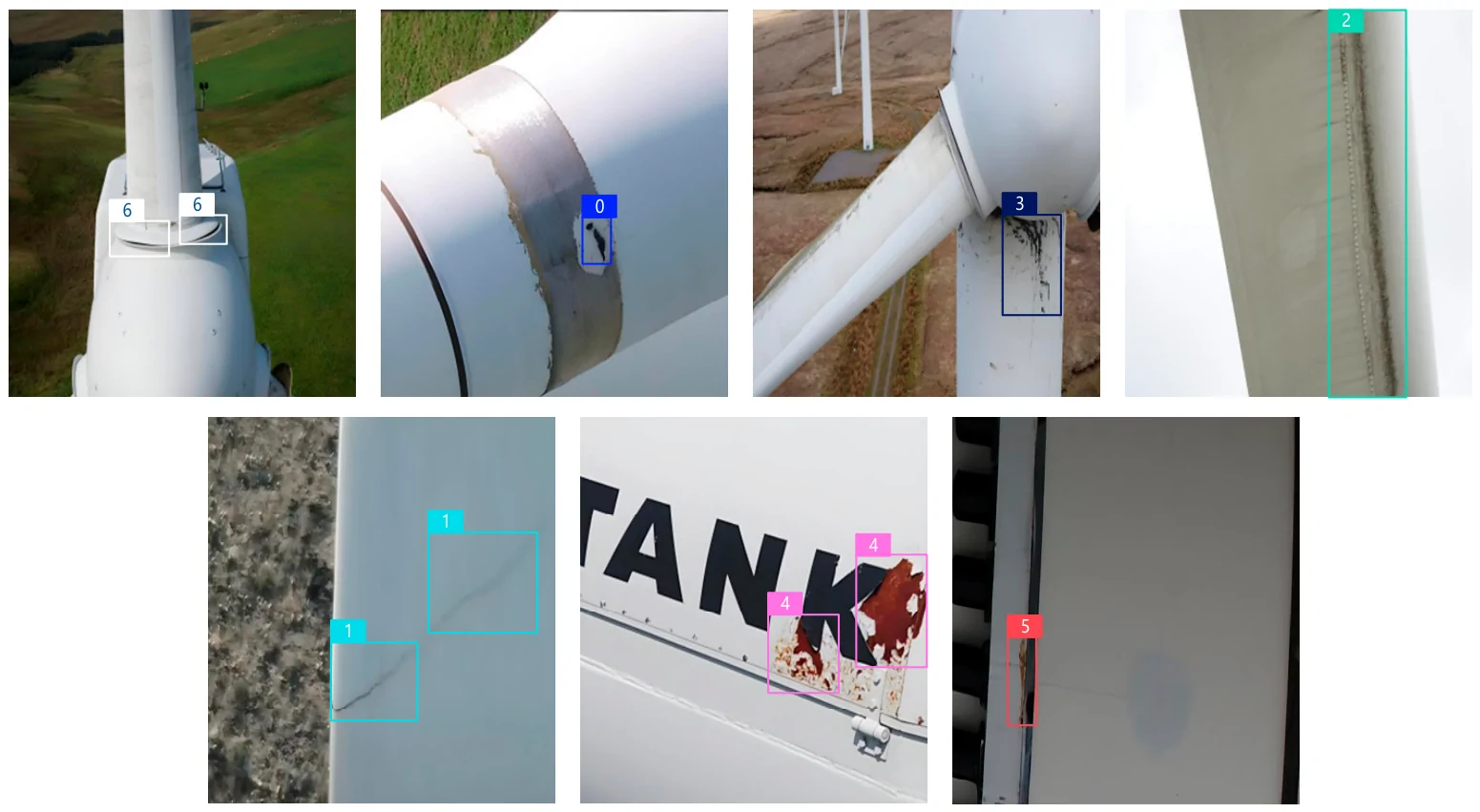
Typical defect types of wind turbine blades.

**Figure 7:**
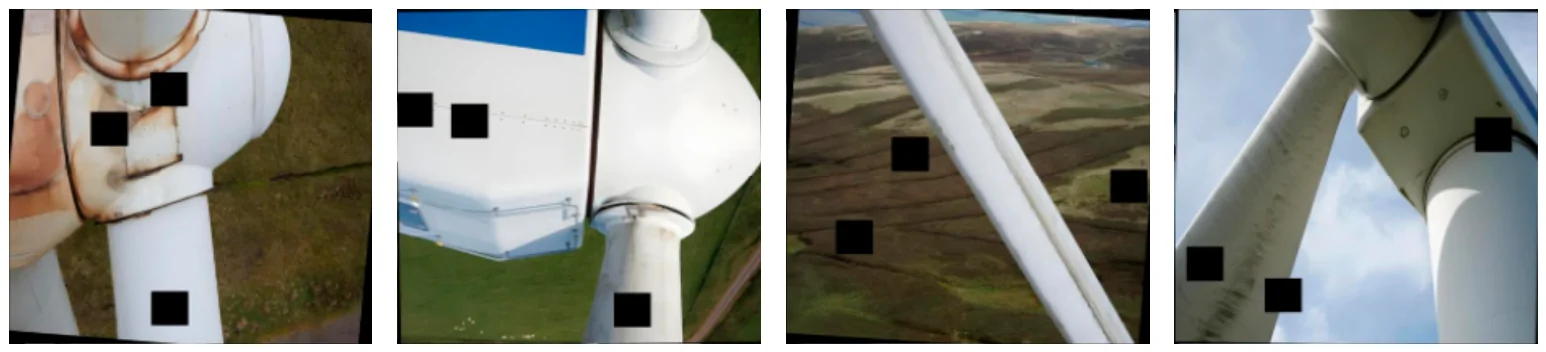
Data augmentation techniques, such as random occlusion and rotation.

To increase sample diversity and improve model adaptability to partial occlusion and viewpoint variations, random occlusion and random rotation were applied for data augmentation during training. This process ultimately yielded the wind turbine defect dataset (WTDD), comprising 7655 images. The dataset was divided into training, validation, and test sets at a ratio of 8:1:1. All comparative models employed the same data partition and augmentation strategies to ensure the fairness and comparability of the experimental results. Representative defect samples and data-augmentation results are presented in Figure 6 and Figure 7, respectively. The raw imagery was captured across multiple flights conducted under diverse weather conditions, and augmentation via random occlusion and rotation (Figure 7) was applied to introduce partial-visibility and viewpoint variability into the training set. Nonetheless, the dataset does not systematically capture motion blur or extreme lighting variation encountered during operational UAV inspection, a limitation discussed further in Section 6.

As shown in Table 1, WTDD exhibits pronounced class imbalance, with an approximate ratio of 7:1 between the largest and smallest classes. To mitigate the dominance of majority classes during training, class-aware repeat sampling is applied to the training set. The sampling weight of class *c* is defined as $\omega_{c}=1/\sqrt{n_{c}}$, where $n_{c}$ denotes the number of instances belonging to class c. For an image containing multiple defect categories, the maximum weight among the corresponding classes is assigned as its image-level sampling weight. This strategy increases the training frequency of minority-class images, while the square-root scaling prevents excessive repetition. The original class distributions are retained in the validation and test sets to ensure objective performance evaluation.

## 5. Results and Discussion

### 5.1. Evaluation Criteria and Experimental Environment Setup

To standardize the evaluation of object detection performance, precision (P), recall (R), F1-score, and mean average precision (mAP) are adopted as the evaluation metrics. Precision represents the proportion of correctly detected defects among all predicted defects, whereas recall represents the proportion of ground-truth defects that are correctly detected. The F1-score provides a comprehensive measure of the balance between precision and recall, while mAP50 evaluates the overall detection performance across all categories at an IoU threshold of 0.50.

(22) $$\mathrm{Precision}=\frac{\mathrm{TP}}{\mathrm{TP}+\mathrm{FP}}$$

(23) $$\mathrm{Recall}=\frac{\mathrm{TP}}{\mathrm{TP}+\mathrm{FN}}$$

(24) $$F1-\mathrm{score}=\frac{2\times \mathrm{Precison}\times \mathrm{Recall}}{\mathrm{Precison}+\mathrm{Recall}}$$

(25) $$\mathrm{mAP}=\frac{1}{N}\sum_{c=1}^{N}\int_{0}^{1}P(R)\mathrm{dR}$$

Herein, TP refers to positive samples correctly predicted as positive. TN denotes negative samples accurately identified as negative. FP is the count of negative samples misclassified as positive. FN indicates positive samples incorrectly predicted as negative. N signifies the number of categories.

The experimental hardware configuration uses an Intel (R) Core i7-14650HX CPU and an NVIDIA GeForce RTX 4060 (16 GB) GPU. The software environment includes the Windows 11 operating system, Python 3.11, PyTorch 2.2.2, and CUDA 12.1.

### 5.2. Discussion on Defect Detection Results

To systematically verify the superior performance of the proposed YOLOv13-ADR model, this study selected five representative advanced object detection algorithms as comparison benchmarks. YOLOv8 [44], serving as a typical lightweight real-time detection model, achieves a balance between computational efficiency and detection accuracy through its decoupled head and anchor-free design. DETR-R50 [45] is employed as a representative Transformer-based detection baseline. The model uses ResNet-50 for feature extraction, followed by a 1 × 1 convolution that projects the extracted features into a 256-dimensional representation. The resulting features are then fed into a six-layer Transformer encoder and decoder. With eight-head attention, 100 object queries, and Hungarian matching, DETR-R50 performs end-to-end object detection without requiring anchor boxes or non-maximum suppression. YOLOv10 [46] innovatively employs spatial-channel decoupled downsampling and large-kernel convolution strategies, leading to a significant enhancement in both detection accuracy and speed. YOLOv12 [47] effectively strengthens the feature extraction capability for small objects through the synergistic design of region attention mechanisms and residual efficient layer aggregation networks (R-ELAN). YOLOv13 [48] integrates hypergraph adaptive correlation enhancement (HyperACE) modules and full-process feature distribution mechanisms (FullPAD), achieving a unification of high-order semantic modeling with model lightweight design. Building upon the advantageous architecture of YOLOv13, the proposed YOLOv13-ADR model in this study enhances the flexibility of sampling positions and shapes via ADConv and enriches contextual information representation by introducing the NNCPR module. Specifically, the designed F-IoU and FA-IoU loss functions significantly improve the bounding box regression accuracy for multi-scale objects, particularly small objects, through an adaptive penalty mechanism. To ensure a fair comparison among the YOLO-series models, all YOLO models were trained using stochastic gradient descent (SGD) with momentum. Unlike Adam and AdamW, SGD does not rely on adaptive learning-rate estimation for parameter updates. Under identical training epochs, learning rates, and regularization settings, this configuration reduces the confounding influence of optimizer differences on architectural performance comparisons and contributes to stable model training. This setting is intended to standardize the optimization conditions across the YOLO-series models and does not imply that SGD is universally superior to Adam or AdamW. DETR-R50 follows the commonly adopted training configuration for Transformer-based detectors and is optimized using AdamW. The detailed architectural parameters and implementation specifics of the compared methods are presented in Table 2.

**Figure 8:**
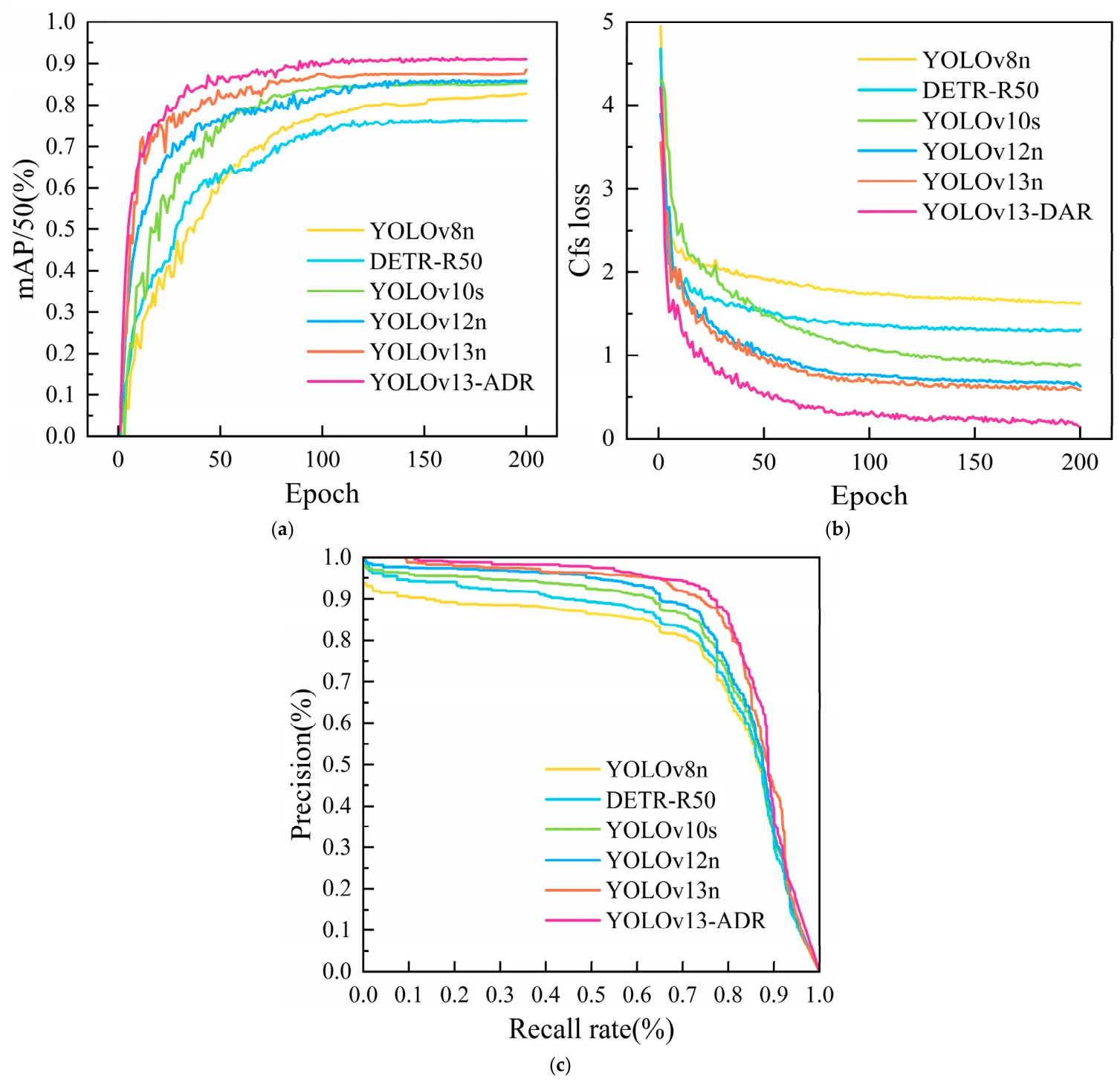
Training and detection performance of the compared methods: (**a**) mAP50 versus training epoch. (**b**) normalized classification–loss convergence curve. (**c**) precision–recall curves.

Figure 8 comprehensively compares the training and detection performance of the investigated models using the mAP50 evolution curves, classification–loss convergence curves, and precision–recall curves. Because DETR-R50 and the YOLO-series models adopt different classification objectives, Figure 8b is primarily used to compare their loss-convergence trends rather than their absolute loss values.

As shown in Figure 8a, DETR-R50 converges relatively slowly under the current dataset scale and number of training epochs, ultimately achieving an mAP50 of 78.43%. YOLOv8n, YOLOv10s, and YOLOv12n achieve mAP50 values of 81.08%, 82.71%, and 84.35%, respectively, indicating that spatial-channel decoupling and region-attention mechanisms progressively improve defect feature extraction. With the high-order semantic modeling capability of HyperACE, YOLOv13n further increases mAP50 to 85.58%. In comparison, YOLOv13-ADR achieves the highest mAP50 of 88.34% and exhibits faster and more stable precision convergence.

As shown in Figure 8b, the classification loss of YOLOv13-ADR decreases more rapidly and exhibits smaller fluctuations during the later training stages, demonstrating favorable optimization stability. By contrast, the loss of DETR-R50 decreases relatively slowly, suggesting that its object-query-based set prediction mechanism has not fully converged under the current training budget.

As shown in Figure 8c, the precision–recall curve of YOLOv13-ADR lies consistently above those of the other methods, demonstrating a superior balance between Precision and Recall. This advantage is primarily attributable to the adaptive sampling of irregular defect geometries by ADConv and the effective reconstruction of contextual information and local details by NNCPR, which jointly improve the recognition of fine cracks and small-scale defects. Detailed quantitative results are presented in Table 3.

The high precision achieved by YOLOv13-ADR is primarily attributable to the reduced number of false-positive detections. ADConv strengthens the geometric representation of irregular defects through adaptive sampling, NNCPR further restores local boundary and contextual information, and F-IoU suppresses the interfering gradients generated by low-quality predicted boxes. These mechanisms reduce the erroneous identification of background regions and defect-like patterns as genuine defects. In addition, the WTDD images contain clearly defined inspection targets and follow relatively consistent annotation criteria. Although occlusion and viewpoint variations were expanded through data augmentation, motion blur and extreme illumination conditions have not yet been systematically covered. Therefore, the current dataset conditions are also conducive to achieving relatively high Precision.

**Figure 9:**
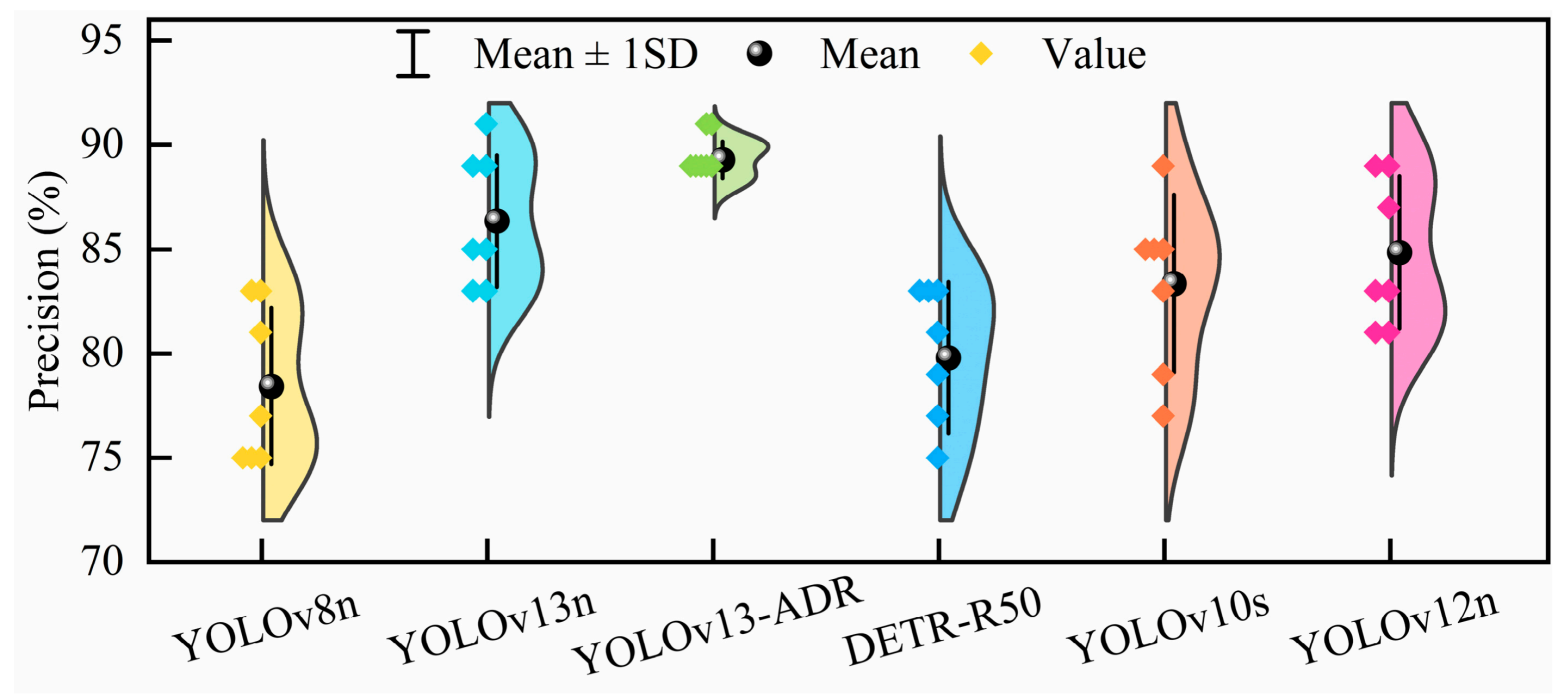
Detection precision of the six comparison methods across the seven defect categories in the WTDD test set.

**Figure 10:**
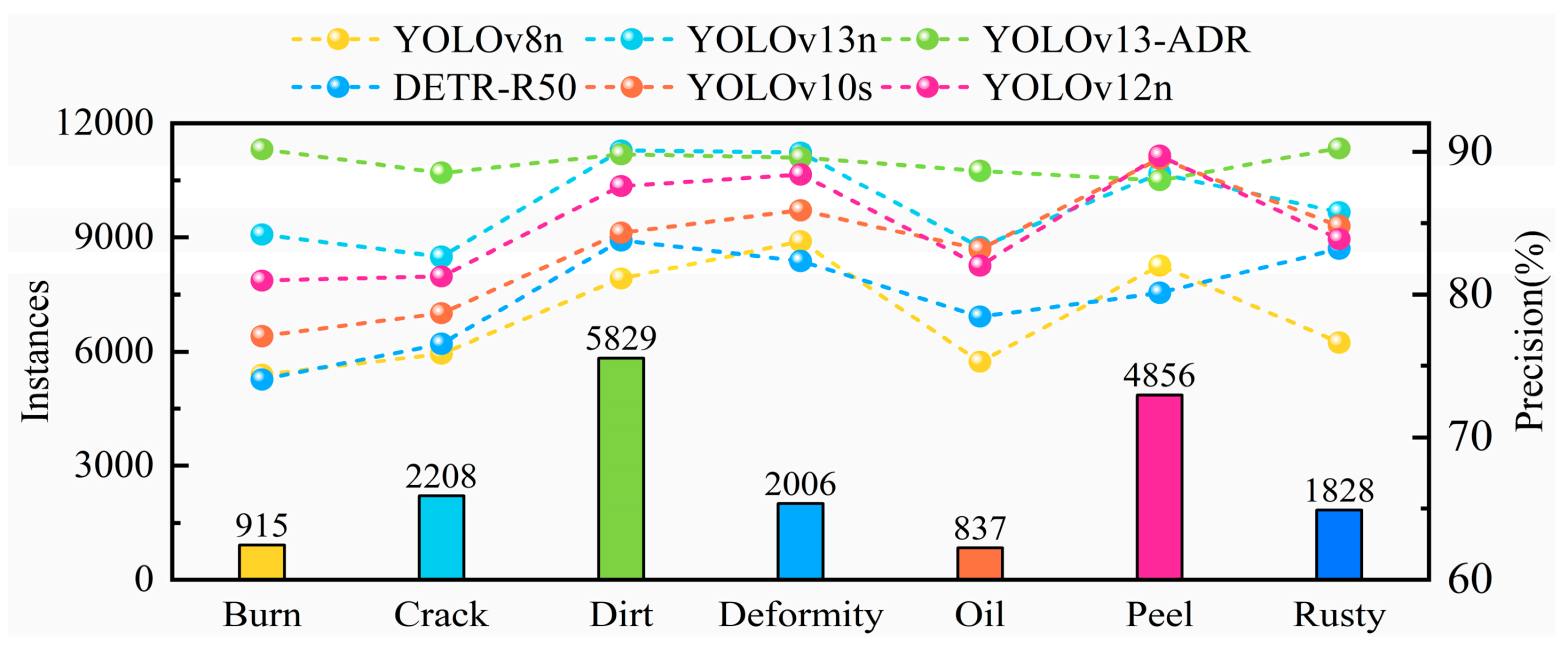
Impact of sample quantity on detection precision for different defect types.

The detection precision for seven representative wind turbine blade defect categories was systematically evaluated, with Figure 9 comparing the performance of the investigated algorithms and Figure 10 quantifying the influence of sample size.

As shown in Figure 9, the YOLOv13-ADR model demonstrated superior performance across all defect categories. However, Figure 10 reveals notable dataset sample distribution imbalance due to significant differences in defect occurrence frequencies in practical scenarios, which affected sample-dependent detection models. Specifically, YOLOv8n’s channel compression strategy limited its feature representation, necessitating abundant samples for compensation. Similarly, DETR-R50 employs a one-to-one set prediction mechanism based on object queries. When the dataset is limited in size and exhibits an imbalanced class distribution, minority-class small-scale defects provide relatively insufficient matching supervision, which may restrict the ability of object queries to fully learn their discriminative features and boundary locations. Consequently, DETR-R50 exhibits more pronounced performance degradation for the underrepresented burn and oil-leakage defects. In comparison, YOLOv12n’s regional attention mechanism enabled feature reuse, reducing instance requirements. YOLOv13n improved feature utilization with HyperACE’s high-order feature associations and FullPAD’s gradient optimization. The proposed YOLOv13-ADR introduces the NNCPR module to adaptively propagate and aggregate contextual features within the convolutional-kernel neighborhood, thereby reducing the model’s dependence on sample quantity and improving its robustness to occluded and incomplete defects. In addition, class-aware repeat sampling is employed during training to increase the participation frequency of minority-class defects. F-IoU further alleviates the optimization bias induced by class imbalance by strengthening the effective regression gradients of small-scale and difficult defects. Through the combined effects of these two strategies, YOLOv13-ADR achieves precision values of 90.17% and 90.24% for the underrepresented burn and oil-contamination categories, respectively, demonstrating favorable detection stability under class-imbalanced conditions. The results of all methods for 7 types of defects are shown in Table 4.

**Figure 11:**
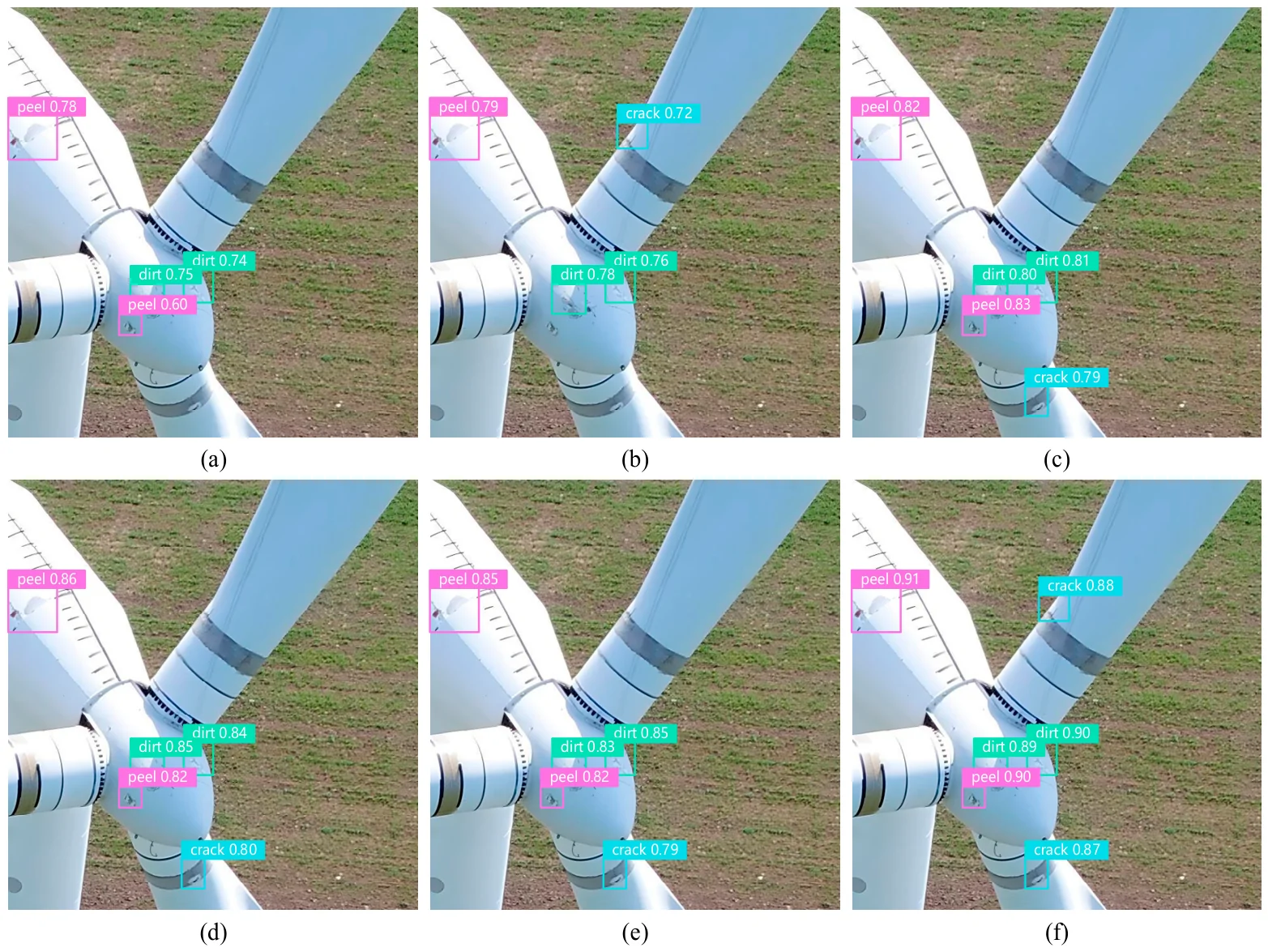
Comparative detection effectiveness of flaking, cracking, and paint stripping defects on wind turbine towers, hubs, and blades. (**a**) YOLOv8n. (**b**) DETR-R50. (**c**) YOLOv10s. (**d**) YOLOv12n. (**e**) YOLOv13n. (**f**) YOLOv13-ADR.

**Figure 12:**
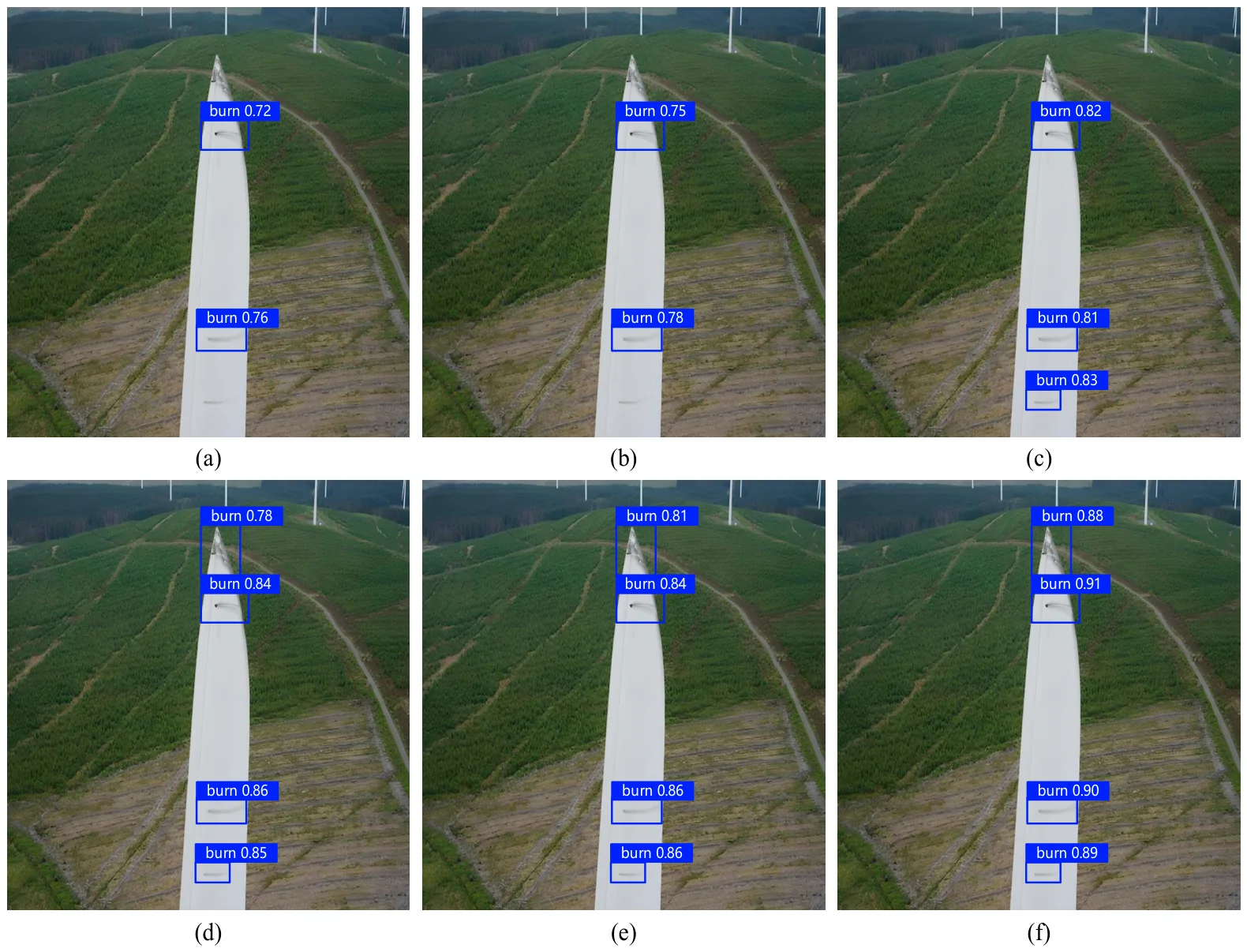
Comparative analysis of burn defect detection on wind turbine blades. (**a**) YOLOv8n. (**b**) DETR-R50. (**c**) YOLOv10s. (**d**) YOLOv12n. (**e**) YOLOv13n. (**f**) YOLOv13-ADR.

**Figure 13:**
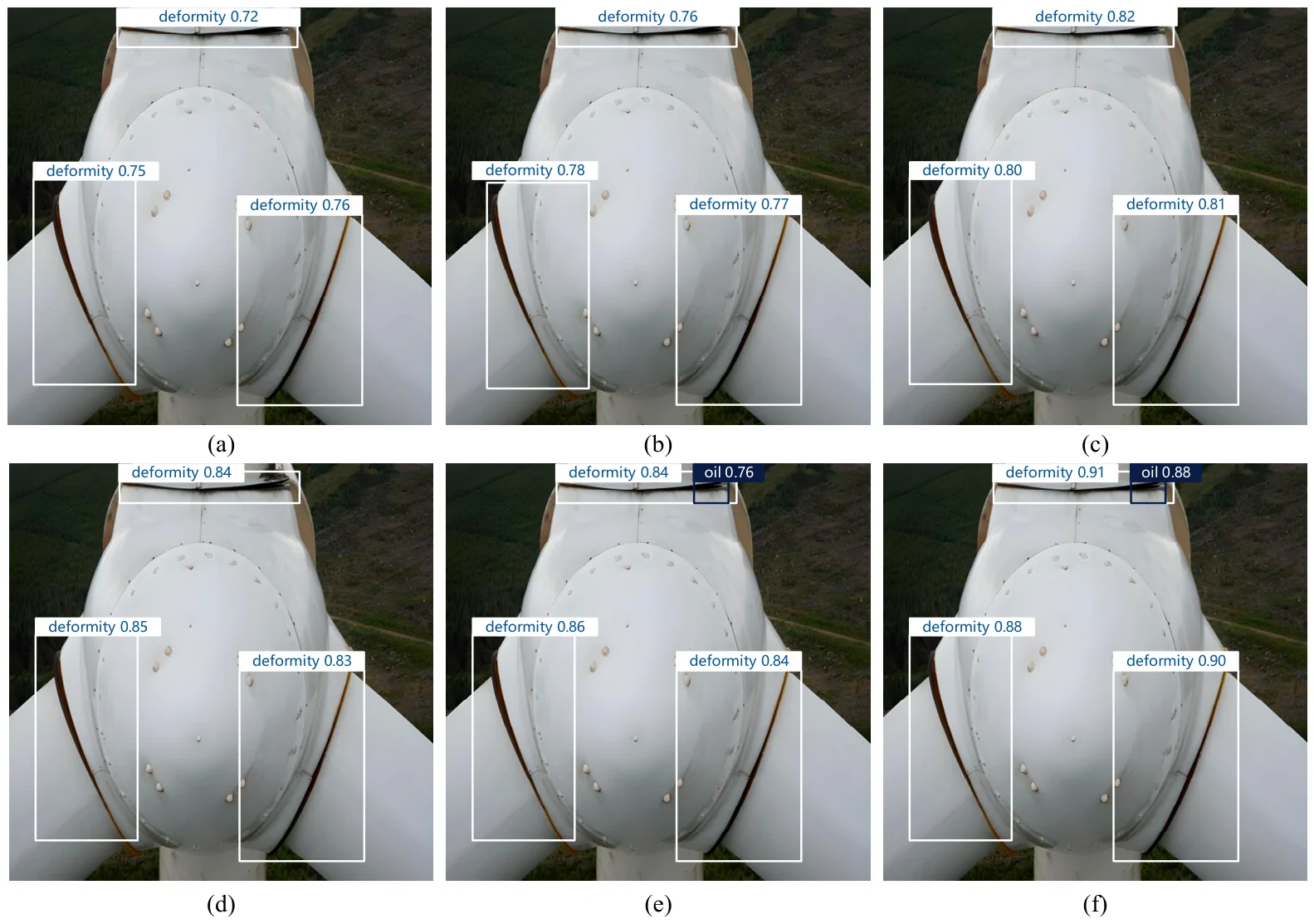
Comparative detection results for deformation and oil-contamination defects in wind turbine hubs. (**a**) YOLOv8n. (**b**) DETR-R50. (**c**) YOLOv10s. (**d**) YOLOv12n. (**e**) YOLOv13n. (**f**) YOLOv13-ADR.

Subsequently, this study conducts a comparative analysis of the detection outcomes for wind turbine structures, including towers, blades, and hubs, across six object detection methods. The detailed detection results are presented in Figure 11, Figure 12 and Figure 13.

As depicted in Figure 11, the wind turbine structures display several typical defects: the tower surface exhibits evident paint stripping, the hub area accumulates significant dirt, and the blades feature minute cracks. A systematic comparative analysis of detection methods (a) through (e) reveals that identifying small-scale targets, such as tower stripping and blade cracks, poses substantial challenges, resulting in varying degrees of missed detections. Specifically, YOLOv8n failed to detect two blade cracks. The DETR-R50 model missed both the tower stripping and a lower blade crack. YOLOv10s, YOLOv12n, and YOLOv13n successfully identified the hub stripping defect but failed to detect the upper blade crack. Furthermore, the detection confidence for small targets was low, with the maximum confidence for the lower blade crack reaching only 0.79. However, the proposed YOLOv13-ADR method demonstrated exceptional small-target detection capabilities. It achieved comprehensive detection of all defects and significantly improved the detection confidence for small targets to 0.90 for paint stripping and 0.88 for blade cracks.

As shown in Figure 12, the study presents a comparative analysis of the detection effectiveness of burn defects on wind turbine blades across all methods. The results indicate that YOLOv8n and DETR-R50 exhibit the most severe cases of missed detections, with two instances each, while YOLOv10s follows with one missed detection. YOLOv12n and YOLOv13n demonstrate lower degrees of missed detections, successfully identifying all burn defects but requiring confidence improvements. YOLOv13-ADR, leveraging its F-IoU loss function that increases penalties for prediction deviations in small targets, assigns higher gradient weights to small or occluded targets with low IoU values, effectively reducing missed detections. In the results depicted in Figure 12f, YOLOv13-ADR achieves detection confidence levels of 0.88, 0.91, 0.90, and 0.89 for the four blade burn defects.

As illustrated in Figure 13, the wind turbine hub has multiple deformation defects and an occluded oil-contamination defect. Methods (a)–(d) failed to detect the oil-contamination defect. YOLOv13n (e) achieved a detection confidence of 0.76, while YOLOv13-ADR (f) demonstrated robust detection of the occluded target with a high confidence level.

### 5.3. Exploration Experiment of Initial Sampling Shape

**Figure 14:**
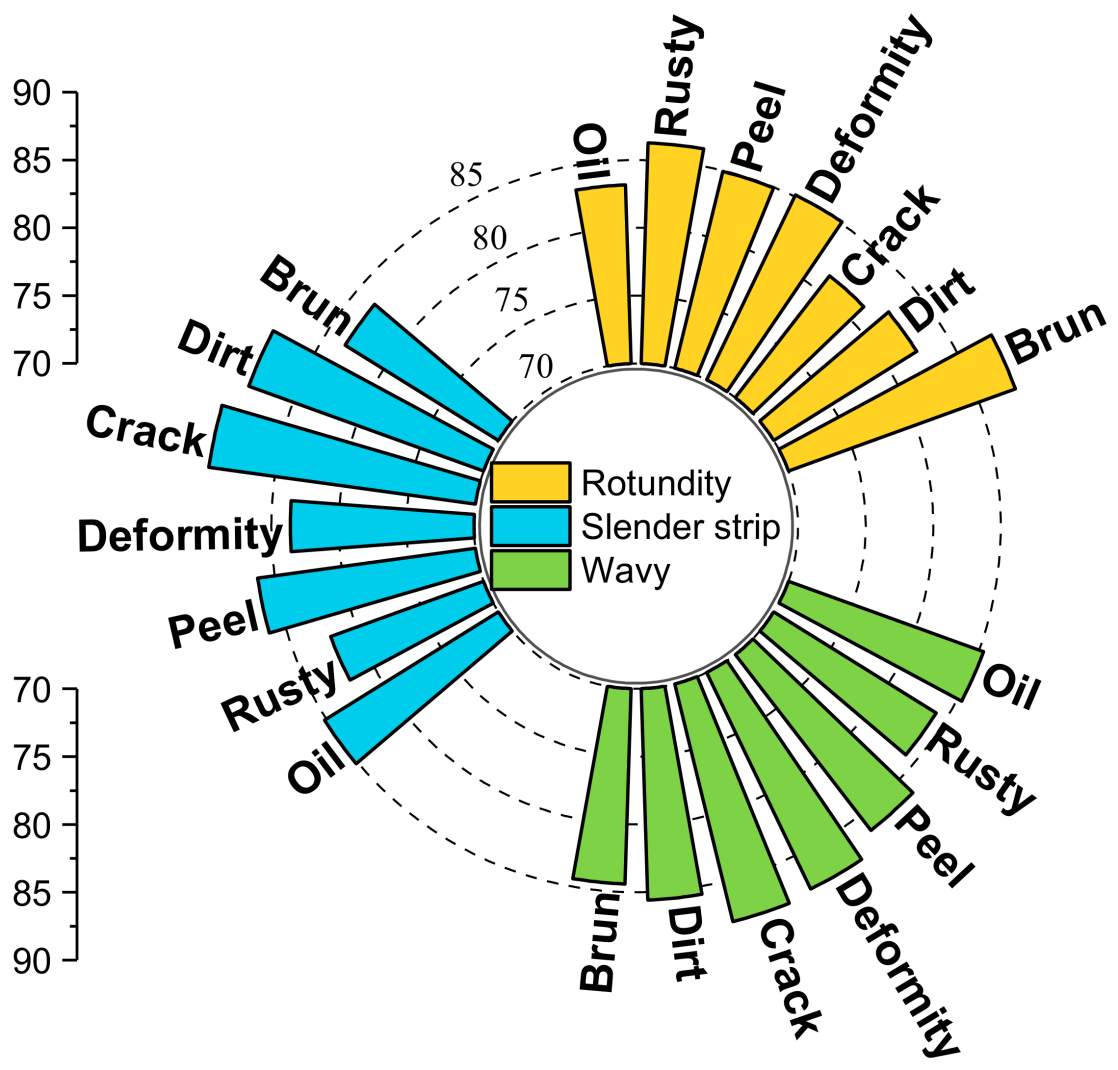
Impact of initial sampling configurations on detection precision for various defect types.

The impact of initial sampling geometries (circular, linear, wavy) on irregular target detection was systematically examined in this research, as presented in Figure 14.

As shown in Figure 14, the experimental data indicates that circular sampling fields are optimal for detecting circular or elliptical defects like burns, deformations, and stripping. This is due to the high geometric match between the sampling shape and these defect types. In contrast, for defects with significant anisotropic features, such as cracks, dirt, and fuel leaks, the detection precision is reduced under circular sampling. However, linear sampling enhances the detection precision of cracks to its peak, which confirms the advantage of linear receptive fields in representing linear features. Notably, wavy sampling shows poor generalization, only specifically improving the detection of stripping, deformation, and cracks.

In summary, the experimental results reveal that the matching degree between sampling shape and target geometric features is a key factor affecting detection precision. The adaptive sampling shape adjustment mechanism achieved by the ADConv module can effectively address the limitations of traditional fixed sampling patterns in complex scenarios. This finding provides an important theoretical basis for constructing intelligent detection systems for multi-shaped targets and is particularly valuable in industrial defect detection applications where multiple geometric features need to be processed simultaneously.

### 5.4. Generalization Performance Evaluation of YOLOv13-ADR

To comprehensively evaluate the generalization of the YOLOv13-ADR, this study conducted cross-domain validation using the NEU-DET steel surface defect dataset from Northeastern University while keeping experimental conditions and hyperparameters consistent. This dataset comprises 4527 high-resolution images with six typical industrial defect categories: Crack, Inclusion, Patches, Pitted Surface, Rolled in Scale, and Scratches. It is divided into 3212 training images, 805 testing images, and 510 validation images, with a sample distribution reflecting real-world industrial inspection scenarios. The specific results are shown in Table 5.

The table indicates that, within the domain of steel surface defect detection, YOLOv13-ADR attains an overall mAP50% of 83.10%, outperforming YOLOv12n (77.85%) and YOLOv13n (79.68%), with the highest individual AP50 values across most defect categories, including Inclusion (84.36%) and Pitted Surface (83.16%). This demonstrates YOLOv13-ADR’s strong generalization capability and suitability for defect detection datasets across diverse scenarios. While this cross-domain evaluation demonstrates generalization across differing material and texture domains, it does not emulate flight-induced degradation such as motion blur or variable illumination present in field UAV inspection, which remains an open validation target discussed in Section 7.

It should be noted that WTDD and NEU-DET do not constitute repeated validation on homogeneous datasets, and the generalization conclusions are restricted to industrial surface-defect detection scenarios. WTDD was collected through UAV-based inspections of wind turbine blades and primarily represents typical defects on composite-material surfaces. In contrast, NEU-DET is a publicly available benchmark dataset for steel-surface defect detection and contains six representative defect categories. The two datasets differ substantially in material type, acquisition mode, background distribution, and defect morphology. They are therefore used to evaluate the effectiveness of the model in the target application scenario and its cross-domain adaptability to heterogeneous industrial environments, respectively. Under unified experimental settings, YOLOv13-ADR consistently outperforms the comparative methods on both datasets. These results indicate that the proposed method is not specifically optimized for a single material type or a particular data distribution, but exhibits favorable generalization capability across different industrial surface defects.

### 5.5. Ablation Experiments and Computational Cost Analysis

To further quantify the individual contributions of ADConv, NNCPR, and F-IoU and evaluate the computational overhead introduced by the additional modules, single-factor ablation experiments are conducted using YOLOv13n as the baseline. B0 denotes the original YOLOv13n, whereas A1 represents the complete YOLOv13-ADR. In A2, ADConv is replaced with the original DS-C3k2 module while the remaining components are retained. In A3, NNCPR is replaced with the original upsampling operation. In A4, F-IoU is replaced with CIoU. All variants employ identical dataset partitions, training parameters, and evaluation settings.

Detection performance is evaluated using Precision, whereas model complexity and computational efficiency are assessed in terms of parameter count, GFLOPs, and single-image inference latency. Precision and inference latency are obtained from five independent experiments and reported as the mean ± standard deviation. The parameter count and GFLOPs are calculated from the fixed network architectures. Inference latency is measured on an RTX 4060 GPU using an input resolution of 640 × 640 and a batch size of 1.

**Figure 15:**
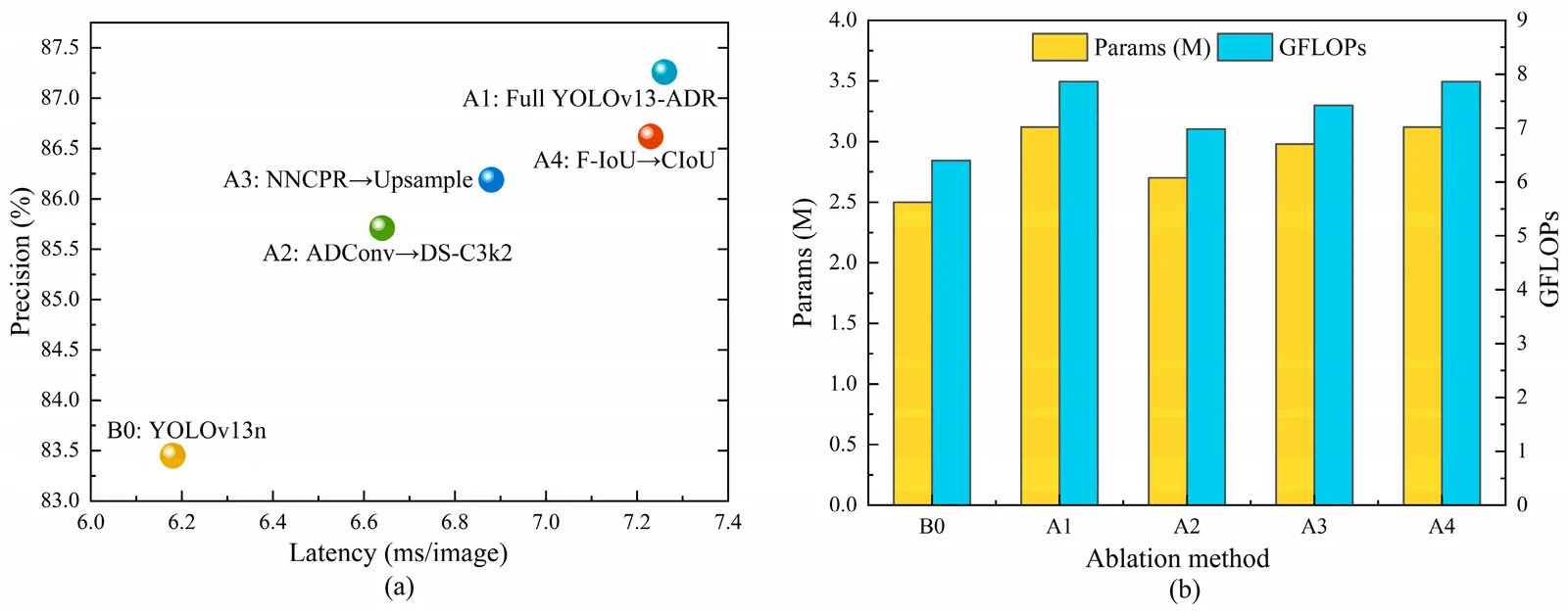
Performance and computational cost analysis of different ablation variants. (**a**) Precision–latency trade-off. (**b**) Parameter count and GFLOPs.

As shown in Figure 15a, the complete YOLOv13-ADR achieves the highest Precision. The Precision values of A2, A3, and A4 are all lower than that of the complete model, indicating that ADConv, NNCPR, and F-IoU each contribute effectively to detection performance. Figure 15b further compares the parameter counts and GFLOPs of the different variants, providing an intuitive illustration of the relationship between performance improvement and computational overhead. Detailed results are presented in Table 6.

Compared with YOLOv13n, the complete YOLOv13-ADR improves Precision by 3.81 percentage points. Replacing ADConv with DS-C3k2 reduces Precision by 1.55 percentage points, indicating that adaptive sampling makes the primary contribution to the extraction of geometric features from irregular defects. Removing NNCPR results in a decrease of 1.07 percentage points, demonstrating that content-aware neighborhood recombination enhances the representation of local boundaries and contextual information. Replacing F-IoU with CIoU reduces Precision by 0.64 percentage points, validating the effectiveness of the scale-adaptive penalty and anchor-quality-aware mechanism.

In terms of computational cost, the complete model introduces an additional 0.62 M parameters and 1.46 GFLOPs compared with YOLOv13n, while the single-image inference latency increases from 6.18 ms to 7.26 ms. ADConv constitutes the primary source of the additional computational cost, whereas NNCPR introduces only limited overhead. Because F-IoU is involved only in loss calculation during training, A1 and A4 have identical parameter counts and GFLOPs, and their difference in inference latency remains within the range of experimental variation. Overall, YOLOv13-ADR achieves a consistent improvement in detection performance while retaining millisecond-level inference efficiency.

## 6. Conclusions

This study proposed YOLOv13-ADR, an enhanced object detection framework for wind turbine blade defect detection, integrating Adaptive Deformable Convolution (ADConv), a Nearest Neighbor Content Perception Recombination (NNCPR) module, and a Focus-IoU loss function within the YOLOv13 architecture. Detailed performance comparisons against five baseline methods, along with cross-domain validation on the NEU-DET steel surface defect dataset, are presented in Section 5 (Table 3, Table 4 and Table 5).

The results indicate that replacing fixed-geometry convolution and content-agnostic upsampling with geometry-adaptive alternatives yields measurable gains specifically for small, irregularly shaped, and occluded defects, which are the categories where conventional YOLO variants and two-stage detectors are most prone to missed detections (Figure 11, Figure 12 and Figure 13). This suggests that geometry-adaptive feature extraction is a promising direction for defect classes defined primarily by irregular morphology rather than size or texture alone, a distinction potentially relevant to structural defect detection tasks beyond wind turbine blades.

The generalization demonstrated to the NEU-DET steel surface dataset (Section 5.4) indicates that the proposed modules are not narrowly tuned to wind turbine blade imagery, supporting potential applicability to other industrial surface inspection tasks where defect geometry is similarly irregular, such as steel structural components and other composite infrastructure.

The improvements demonstrated in detection precision under occlusion and variable defect scale suggest potential applicability to condition-based inspection strategies in offshore wind operations, where inspection access is constrained by weather windows and vessel availability. This is contingent on future validation of inference speed and precision on embedded UAV hardware, as discussed in Section 7, since all reported results in this study were obtained on the desktop GPU configuration specified in Section 5.1.

## 7. Limitations and Future Work

Despite the demonstrated performance gains, several limitations warrant consideration. The WTDD is limited in scale and was collected under near-controlled imaging conditions; although random occlusion and rotation augmentation (Section 4) and cross-domain validation on NEU-DET (Section 5.4) provide preliminary evidence of robustness, performance under field UAV conditions, including rain, fog, and haze-induced contrast loss, motion blur from platform vibration, sun glare, and low-light sensor noise, has not been systematically evaluated. These factors are expected to particularly affect low-contrast defects such as hairline cracks and the boundary reconstruction accuracy of the NNCPR module. The current framework also operates on static images and does not address real-time inference constraints on offshore edge hardware, and the proposed modifications increase computational parameters relative to the YOLOv13n baseline, which may limit deployment on resource-constrained systems. Future work should prioritize a field-acquired dataset spanning these degradation conditions, evaluation under onshore–offshore domain shift, integration with UAV autonomous flight control for end-to-end inspection, and lightweight compression and quantization for real-time edge deployment.

## Figures and Tables

**Table 1:** Number of instances across the seven wind turbine blade defect categories.

F0	F1	F2	F3	F4	F5	F6
Burn	Crack	Dirt	Oil	Peel	Rusty	Deformity
915	2208	5829	837	4856	1828	2006

**Table 2:** Descriptions and parameter settings of comparison methods.

Method	Epoch	Batch Size	Optimizer	Learning Rate	Momentum	Weight Decay
YOLOv8n	200	32	SGD	0.01	0.825	0.002
DETR-R50	200	32	AdamW	0.01	-	0.002
YOLOv10s	200	32	SGD	0.01	0.825	0.002
YOLOv12n	200	32	SGD	0.01	0.825	0.002
YOLOv13n	200	32	SGD	0.01	0.825	0.002
YOLOv13-ADR	200	32	SGD	0.01	0.825	0.002

**Table 3:** Overall performance of comparative methods.

Method	P (%)	R (%)	F1 Score (%)	mAP50 (%)
YOLOv8n	81.11 ± 0.47	80.25 ± 0.55	80.68 ± 0.52	81.08 ± 0.42
DETR-R50	78.34 ± 0.74	77.18 ± 0.71	77.76 ± 0.53	78.43 ± 0.63
YOLOv10s	82.56 ± 0.45	81.42 ± 0.48	81.99 ± 0.51	82.71 ± 0.36
YOLOv12n	85.02 ± 0.38	83.76 ± 0.40	84.39 ± 0.40	84.35 ± 0.31
YOLOv13n	83.45 ± 0.36	84.37 ± 0.34	83.91 ± 0.36	85.58 ± 0.28
YOLOv13-ADR	87.26 ± 0.31	88.63 ± 0.30	87.94 ± 0.30	88.34 ± 0.24

**Table 4:** Precision of comparative methods for seven defect types.

Method	Precision (%)						
	Burn	Crack	Dirt	Deformity	Peel	Rusty	Oil
YOLOv8n	74.37 ± 0.78	75.85 ± 0.82	81.12 ± 0.65	83.72 ± 0.61	75.29 ± 0.76	82.02 ± 0.64	76.63 ± 0.73
DETR-R50	74.04 ± 0.91	76.55 ± 0.88	83.78 ± 0.72	82.35 ± 0.79	78.46 ± 0.84	80.11 ± 0.87	83.21 ± 0.75
YOLOv10s	77.07 ± 0.68	78.68 ± 0.72	84.33 ± 0.57	85.89 ± 0.55	83.15 ± 0.61	89.52 ± 0.49	84.80 ± 0.58
YOLOv12n	80.97 ± 0.56	81.26 ± 0.61	87.59 ± 0.47	88.42 ± 0.45	82.03 ± 0.59	89.71 ± 0.43	83.88 ± 0.54
YOLOv13n	84.19 ± 0.49	82.64 ± 0.58	90.09 ± 0.42	89.95 ± 0.40	83.31 ± 0.55	88.48 ± 0.46	85.76 ± 0.51
YOLOv13-ADR	90.17 ± 0.38	88.53 ± 0.46	89.82 ± 0.37	89.58 ± 0.39	88.67 ± 0.44	88.01 ± 0.48	90.24 ± 0.35

**Table 5:** Testing results of YOLOv13-ADR on the NEU-DET steel surface defect dataset.

Method	AP50 (%)						mAP50 (%)
	Crack	Inclusion	Patches	Scratches	Pitted Surface	Mill Scale	
YOLOv12n	75.19 ± 0.64	76.33 ± 0.59	78.18 ± 0.55	80.52 ± 0.48	79.76 ± 0.52	77.16 ± 0.61	77.85 ± 0.37
YOLOv13n	77.35 ± 0.56	78.91 ± 0.52	78.32 ± 0.58	80.06 ± 0.47	81.35 ± 0.45	82.09 ± 0.43	79.68 ± 0.33
YOLOv13-ADR	79.21 ± 0.49	84.36 ± 0.38	82.08 ± 0.42	82.58 ± 0.39	83.16 ± 0.36	81.23 ± 0.51	83.10 ± 0.29

**Table 6:** Ablation results and computational costs of YOLOv13-ADR.

Variant	Configuration	Precision (%)	Latency (ms/Image)	Params (M)	GFLOPs
B0	YOLOv13n	83.45 ± 0.36	6.18 ± 0.11	2.50	6.40
A1	Full YOLOv13-ADR	87.26 ± 0.31	7.26 ± 0.13	3.12	7.86
A2	ADConv → DS-C3k2	85.71 ± 0.35	6.64 ± 0.12	2.70	6.98
A3	NNCPR → Upsample	86.19 ± 0.33	6.88 ± 0.11	2.98	7.42
A4	F-IoU → CIoU	86.62 ± 0.32	7.23 ± 0.12	3.12	7.86

## Data Availability

The datasets presented in this article are not readily available due to technical and time limitations. Requests to access the datasets should be directed to wangxinwei@ncwu.edu.cn.
